# Regulatory changes in *TaSNAC8‐6A* are associated with drought tolerance in wheat seedlings

**DOI:** 10.1111/pbi.13277

**Published:** 2019-11-19

**Authors:** Hude Mao, Shumin Li, Zhongxue Wang, Xinxiu Cheng, Fangfang Li, Fangming Mei, Nan Chen, Zhensheng Kang

**Affiliations:** ^1^ State Key Laboratory of Crop Stress Biology for Arid Areas College of Plant Protection Northwest A&F University Yangling Shaanxi China

**Keywords:** wheat, NAC transcription factor, *TaSNAC8‐6A*, natural variation, lateral root development, drought stress

## Abstract

Wheat is a staple crop produced in arid and semi‐arid areas worldwide, and its production is frequently compromised by water scarcity. Thus, increased drought tolerance is a priority objective for wheat breeding programmes, and among their targets, the NAC transcription factors have been demonstrated to contribute to plant drought response. However, natural sequence variations in these genes have been largely unexplored for their potential roles in drought tolerance. Here, we conducted a candidate gene association analysis of the stress‐responsive *NAC* gene subfamily in a wheat panel consisting of 700 varieties collected worldwide. We identified a drought responsive gene, *TaSNAC8‐6A*, that is tightly associated with drought tolerance in wheat seedlings. Further analysis found that a favourable allele *TaSNAC8‐6A* ^In‐313^, carrying an insertion in the ABRE promoter motif, is targeted by TaABFs and confers enhanced drought‐inducible expression of *TaSNAC8‐6A* in drought‐tolerant genotypes. Transgenic wheat and *Arabidopsis TaSNAC8‐6A* overexpression lines exhibited enhanced drought tolerance through induction of auxin‐ and drought‐response pathways, confirmed by transcriptomic analysis, that stimulated lateral root development, subsequently improving water‐use efficiency. Taken together, our findings reveal that natural variation in *TaSNAC8‐6A* and specifically the *TaSNAC8‐6A* ^In‐313^ allele strongly contribute to wheat drought tolerance and thus represent a valuable genetic resource for improvement of drought‐tolerant germplasm for wheat production.

## Introduction

Drought has caused a 13.7 % average loss in worldwide cereal production over recent decades (Lesk *et al.*, 2016; Pennisi, 2008). Among these cereals, wheat (*Triticum aestivum* L.) is an important staple crop, mainly grown in arid and semi‐arid regions worldwide. Its production is frequently compromised by water scarcity, which is exacerbated by the trends of climate warming and population growth on a global scale (Lesk *et al.*, 2016). Therefore, improvement of drought‐tolerant germplasm for higher yields in arid cultivation regions is therefore an important goal among wheat breeding programmes. However, drought tolerance is a complex trait controlled by many genes with minor effects. Several previously identified genes necessary for drought tolerance have been characterized for their involvement in signal perception, signal transduction and transcriptional regulation of other genes necessary for drought tolerance, including protein kinases and transcription factors (TFs), such as members of the AP2/ERF, MYB, bZIP and NAC TF families (Hu and Xiong, 2014; Liu *et al.*, 2014; Nakashima *et al.*, 2012). TFs are particularly attractive targets for genetic improvement of abiotic stress tolerance in crops because the engineering of a single TF can affect all of the genes in its respective regulon, consequently impacting the physiological process or trait for which they are responsible (Gahlaut *et al.*, 2016; Hu and Xiong, 2014).

The NAC (NAM, ATAF1/2 and CUC2) proteins comprise a large family of plant‐specific TFs whose members participate in a range of regulatory and developmental processes, including growth and differentiation at the shoot apical meristem (Takada *et al.*, 2001), root development (Xie *et al.*, 2000), leaf senescence (Yang *et al.*, 2011), formation of secondary cell walls (Mitsuda *et al.*, 2007), nutrient remobilization to grains (Uauy *et al.*, 2006) and abiotic stress response (Nakashima *et al.*, 2012; Shao *et al.*, 2015). Many stress‐responsive NAC proteins fall into one group (Nakashima *et al.*, 2012; Nuruzzaman *et al.*, 2010), such as *ATAF1*, *ANAC019*, *ANAC055*, *ANAC072*, *AtNAC2* and *AtNAP* from *Arabidopsis* (He *et al.*, 2005; Sakuraba *et al.*, 2015; Tran *et al.*, 2004; Wu *et al.*, 2009); *SNAC1*, *OsNAC5*, *OsNAC6*, *OsNAC10* and *OsNAP* from rice (Chen *et al.*, 2014; Hu *et al.*, 2006; Jeong *et al.*, 2010; Jeong *et al.*, 2013; Lee *et al.*, 2017); and *ZmSNAC1*, *ZmNAC55* and *ZmNAC111* from maize (Lu *et al.*, 2012; Mao *et al.*, 2015; Mao *et al.*, 2016).

Notably, a number of these stress‐responsive NAC TFs have been extensively validated through transgenic overexpression as a means to increase crop drought tolerance and to improve yield or biomass. For example, overexpression of *SNAC1* in rice or wheat led to significantly enhanced drought tolerance and yield under severe drought conditions without any observable phenotypic changes or yield penalty under well‐watered growth conditions (Hu *et al.*, 2006; Saad *et al.*, 2013); elevating the expression of *OsNAC5*, *OsNAC6* and *OsNAC10* in rice can also improve drought tolerance as well as grain yield under drought conditions (Jeong *et al.*, 2010; Jeong *et al.*, 2013; Lee *et al.*, 2017). In addition, overexpression of *OsNAC5*, *OsNAC6* and *OsNAC10* in rice roots induces radial root growth, which is a key adaptation to drought stress through root growth and development in response to water‐deficit conditions (Jeong *et al.*, 2010; Jeong *et al.*, 2013; Lee *et al.*, 2017). Thus, previous research has established that stress‐responsive *NAC* TFs can serve as useful candidates for improvement of stress‐tolerant crop germplasm without introducing detrimental growth defects.

However, as of yet no quantitative trait loci (QTL) responsible for drought tolerance in wheat have been cloned, despite some reports of mapping information (Fleury *et al.*, 2010; Gahlaut *et al.*, 2017; Quarrie *et al.*, 1994). In this study, we used linkage disequilibrium (LD)‐based association mapping, a powerful tool for dissecting complex agronomic traits and identifying alleles that can contribute to crop improvement, in conjunction with candidate gene association analysis for detecting DNA polymorphisms (e.g. SNPs) or genetic markers within, or tightly linked to genes contributing to complex traits (Huang *et al.*, 2011; Liu *et al.*, 2017; Wang and Qin, 2017; Xiao *et al.*, 2017). This approach has previously been successfully applied to detect allelic diversity of genes controlling crop drought tolerance, especially in rice and maize (Liu *et al.*, 2013; Mao *et al.*, 2015; Singh *et al.*, 2015; Songyikhangsuthor *et al.*, 2014; Wang *et al.*, 2016; Xiang *et al.*, 2017; Xiong *et al.*, 2018).

Using the IWGSC, 2018 wheat genome (IWGSC, 2018), we identified and characterized 39 *TaSNAC* genes, which formed 13 homologous groups in wheat. The association between genetic variations within each candidate *TaSNAC* gene and drought tolerance, evaluated in terms of survival rate (SR) under severe drought stress at the seedling stage, was quantified using a candidate gene association strategy and a diversity panel consisting of 700 wheat varieties from global germplasm. A strong association was detected between *TaSNAC8‐6A* and SR in the seedling stage. Further analysis showed that sequence variation in the promoter region of *TaSNAC8‐6A*, specifically an indel in the ABRE motif, was associated with distinct patterns of gene expression in response to drought stress across several different wheat varieties. Additionally, overexpression of *TaSNAC8‐6A* up‐regulated several putative NAC‐targeted genes involved in auxin‐ and drought‐response signalling pathways, subsequently stimulating the development of lateral roots and consequently enhancing tolerance to drought stress.

## Results

### Identification and characterization of *TaSNAC* genes

**Figure 1:**
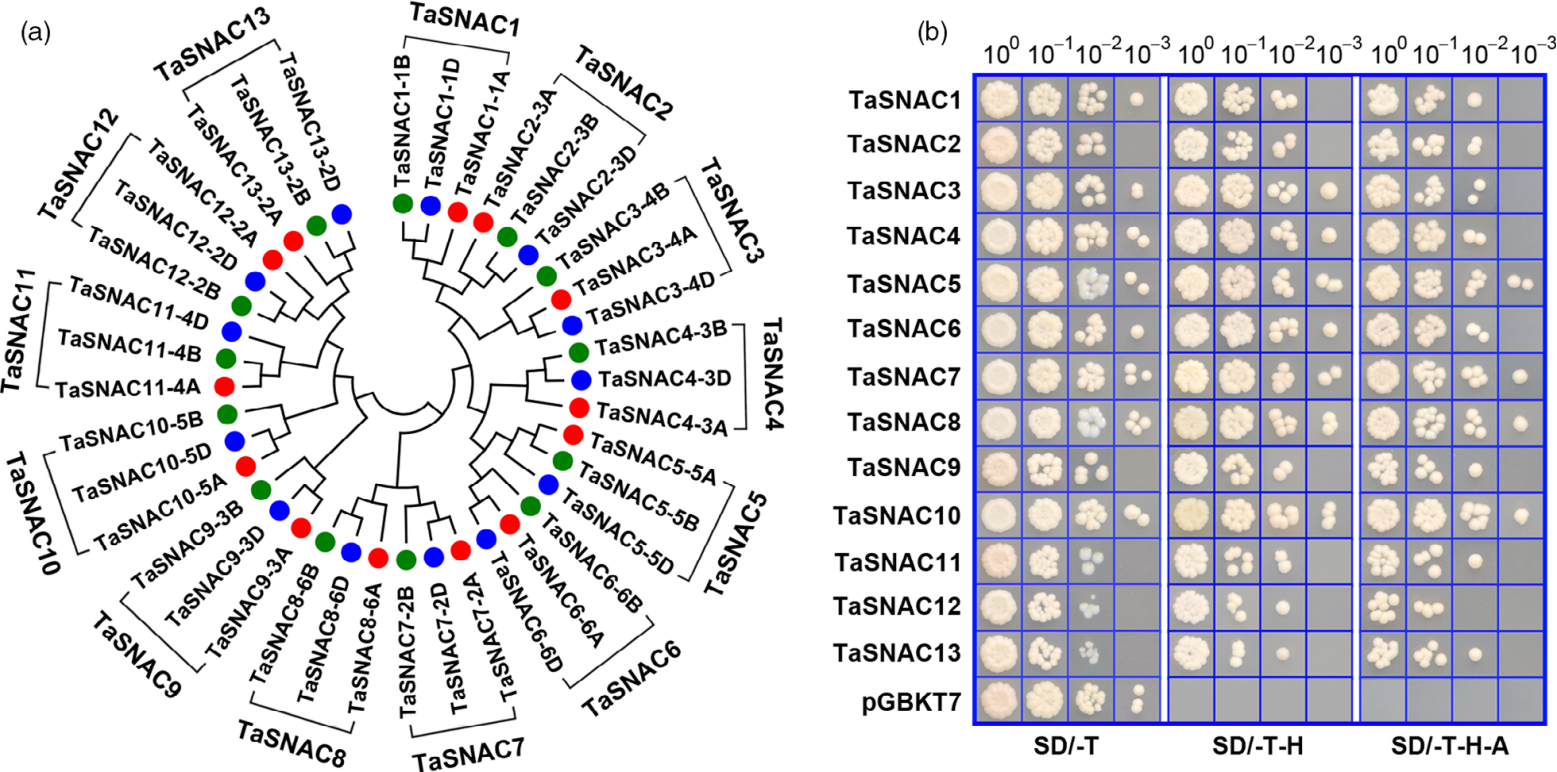
Phylogeny of 13 TaSNAC homologous groups and transactivation activity assay of TaSNAC proteins. (a) Phylogenetic tree of TaSNACs in wheat. 39 TaSNACs were classified into 13 homologous groups designated as TaSNAC1–TaSNAC13, followed by chromosomal locations. (b) Transactivation activity assay of 13 TaSNAC proteins.

In order to identify stress‐responsive NAC subfamily genes in wheat, we conducted a BLAST search against the wheat genome database (IWGSC RefSeq v1.1) using *Arabidopsis* and rice NAC TFs as queries. Returned hits were pooled and redundancies were eliminated. Each sequence was manually examined to confirm NAC domain presence and location. In total, 260 NAC‐domain proteins were identified in the wheat genome (Table S1). Based on *Arabidopsis* and rice NAC superfamily phylogenies (Nakashima *et al.*, 2012), 39 wheat NAC TFs were classified in the stress‐responsive NAC subfamily. Since wheat is hexaploid with three subgenomes (2n = 6x = 42; AABBDD), we further classified these 39 wheat NAC TFs into 13 homologous groups. The three homologous alleles were designated as *TaSNACX_YA*, *TaSNACX_YB* or *TaSNACX_YD*, where X denotes the homologous group number and Y the wheat chromosome where it is located. The previously identified proteins TaNAC2 (Mao *et al.*, 2012) and TaNAC29 (Huang *et al.*, 2015) were renamed as TaSNAC5‐5A and TaSNAC13‐2A, respectively (Figure 1a).

To characterize the phylogenetic relationships between TaSNAC proteins and their orthologs from rice, *B*. *distachyon*, maize and *Arabidopsis*, a neighbour‐joining phylogenetic tree was reconstructed using full‐length protein sequences (Figure S1). These stress‐responsive NAC proteins clustered into two distinct subgroups consistent with previous reports (Nakashima *et al.*, 2012). Several NAC proteins from four monocot species displayed pairwise correspondences with high bootstrap support, suggesting high interspecific conservation. Furthermore, wheat NACs were phylogenetically closer to *B*. *distachyon* than to rice or maize, consistent with the closer evolutionary relationship between wheat and *B*. *distachyon* than with other species in this study. *TaSNAC7‐2A/B/D* and *TaSNAC8‐6A/B/D*, and *TaSNAC12‐2A/B/D* and *TaSNAC13‐2A/B/D* most likely represent duplicated genes in wheat since only one ortholog from each pair was found in the *B*. *distachyon*, rice and maize genomes (Figure S1).

In order to isolate and functionally characterize individual *TaSNAC* genes, we cloned 39 of them from wheat *cv.* Chinese Spring and performed a transactivation activity screen of the encoded proteins using a yeast transactivation assay. Due to the high sequence similarity within groups of homologous proteins (>95%), we subcloned 13 genes, one from each homologous group, into a yeast GAL4‐DNA binding domain expression vector, which was subsequently transformed into yeast reporter cells. The TaSNAC‐GAL4 fusion proteins exhibited a relatively higher level of transactivation activity as demonstrated by their ability to grew well on selective medium (Figure 1b). These genes are distributed across 18 of the wheat chromosomes except chromosomes 7A, 7B and 7D. Gene structure analysis revealed that three *TaSNAC* genes have one exon, seven have two exons, 28 have three exons, and one has four exons (Figure S2).

### Expression profiles of *TaSNAC* genes

**Figure 2:**
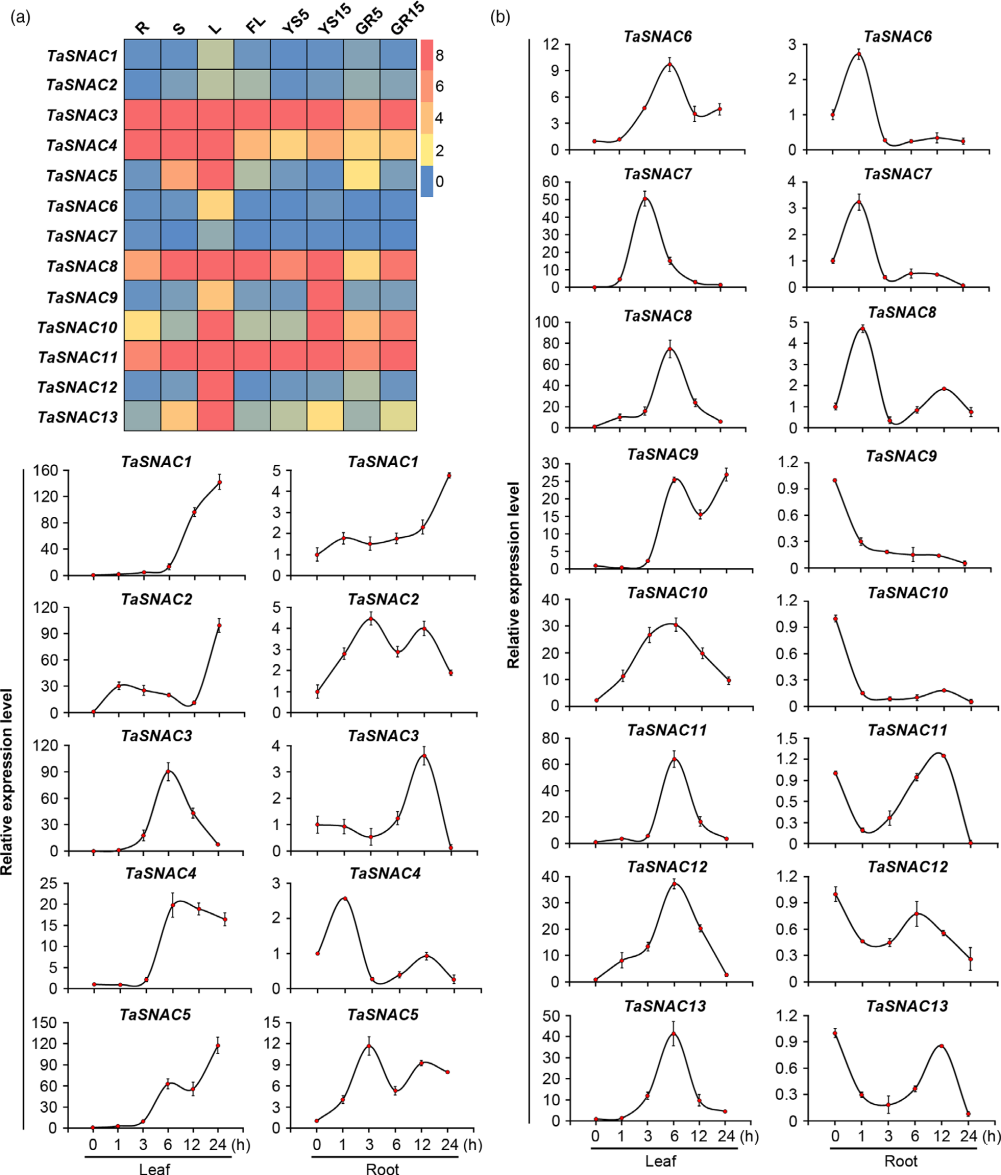
Expression profiles of wheat *TaSNAC* genes. (a) Gene expression heatmap of 13 *TaSNAC* genes in eight different tissues from various developmental stages. R, S, and L, seedling root, stem, and leaf at three‐leaf stage, respectively; FL, flag leaf at heading stage; YS5, young spike at early booting stage; YS15, spike at heading stage; GR5 and GR15, the grain of 5 and 15 days postanthesis, respectively. The gene expression levels are shown in different colours indicated by the scale bar. (b) Relative gene expression levels of 13 *TaSNAC* genes in leaf and root tissue with or without drought stress. Data represent the mean ± SD of three replicates.

In order to better understand the function of each of the *TaSNAC* genes, we first examined *TaSNAC* expression in eight different wheat tissues under normal growth conditions by quantitative real‐time PCR (qRT‐PCR). The PCR primers were designed to amplify the homologous alleles at a conserved locus; for example, the relative expression level of *TaSNAC1* represents the combined expression of all three homologous *TaSNAC1* alleles (*TaSNAC1‐1A*, *TaSNAC1‐1B* and *TaSNAC1‐1D*). The results revealed high variation among the expression patterns of different *TaSNAC* genes (Figure 2a). *TaSNAC1/2/6/7* had relatively low expression levels in the eight tissues, while *TaSNAC3/11* were expressed at relatively high levels. *TaSNAC4/8* were also expressed at relatively high levels in these tissues. However, *TaSNAC4* had higher expression levels in seedling leaves, stems and roots, while *TaSNAC8* had higher expression in seedling leaves and stems, flag leaves, spikes at 15‐DAP (days after pollination) and 15‐DAP grains. The remaining genes were highly expressed only in specific tissues. For example, *TaSNAC5/12/13* were expressed in leaves; *TaSNAC9* was expressed in 15‐DAP spikes; and *TaSNAC10* was expressed at high levels in seedling leaves, 15‐DAP spikes and 15‐DAP grains (Figure 2a).

To better understand the regulatory functions of individual *TaSNAC* gene in response to drought, the expression of 13 *TaSNACs* was examined by qRT‐PCR in leaves and roots of three‐leaf stage seedlings under drought stress. We observed a dramatic up‐regulation of all *TaSNACs* in response to drought, especially in leaf tissue (Figure 2b). The expression of *TaSNAC1* and *TaSNAC5* in leaves after drought stress was increased > 100‐fold, indicating extreme sensitivity to drought. In leaf tissue, some genes exhibited similar expression profiles, such as the two pairs of genes *TaSNAC11/13* and *TaSNAC8*/*12.* Some *TaSNAC*s were up‐regulated after light/early drought stress, such as *TaSNAC2/8/10/12* in leaves and *TaSNAC2/4/5/6/7/9* in roots, suggesting that these genes are transcriptionally activated as part of an early response to drought stress. Other genes were up‐regulated only after severe stress, for example *TaSNAC1* in leaves and roots, suggesting that they participate in the response to severe drought. We also observed sets of genes that are differentially expressed in specific tissues during stress response, such as *TaSNAC8/10/12/13*, which were up‐regulated in leaves but down‐regulated in roots (Figure 2b).

In order to determine which regulatory elements exerted control over *TaSNACs*, we next analysed the DNA sequence of the promoter regions of the 39 *TaSNAC* genes to identify *cis*‐acting regulatory elements involved in the drought stress response, such as ABA‐responsive elements (ABRE), MYB‐binding sites (MYBR) and dehydration‐responsive elements (DRE) (Yamaguchi‐Shinozaki and Shinozaki, 2005). We found that all of the *TaSNAC* genes have one or more of these *cis* elements (Table S2), suggesting stress‐activated regulatory control of these *TaSNAC* genes, and likely a pivotal role in the transcriptional response to drought. Collectively, these data indicate that different *TaSNAC* genes exhibit variation in their expression patterns, dependent on developmental stage, tissue type and drought conditions, thus suggesting diverse roles in developmental regulation during drought stress.

### Association analysis of natural variation in *TaSNAC*s with wheat drought tolerance

Previous studies have reported that stress‐responsive genes *TaSNAC5‐5A* and *TaSNAC13‐2A* participate in regulation of stress response to drought in wheat (Huang *et al.*, 2015; Mao *et al.*, 2012). To further investigate whether genetic variation in any of the *TaSNACs* was associated with phenotypic differences in drought tolerance among varieties, we conducted an association analysis for each of the *TaSNAC* genes. Recently, 630 517 high‐quality wheat SNP markers (660K SNP array) were identified from RNA sequencing or genotyping by sequencing (GBS) of a wheat natural diversity panel consisting of a 110 varieties collected internationally (Rasheed *et al.*, 2017). Using this wheat SNP array, a total of 623 529 high‐quality SNP markers with minor allele frequency equal to or more than 0.05 were identified in a larger, separate wheat panel consisting of a worldwide collection of 700 varieties (Figure S3a,b). These SNP markers were used to identify DNA polymorphisms in each of these 39 *TaSNAC* genes.

Given the estimated LD decay rate of more than 200 kb in wheat varieties (Figure S3c), we selected SNP markers within a 400‐kb interval centred on each *TaSNAC* gene. All of these *TaSNACs* were found to be polymorphic, with an average of 19 SNP markers detected for each *TaSNAC* (Table 1). *TaSNAC8‐6A* was found to be the most polymorphic, with 57 SNPs identified in this natural diversity panel. The drought stress tolerance phenotype of each wheat variety was also investigated, and the survival rate (SR) of seedlings under severe drought conditions was scored (Figure S3d). Then, to assess whether there were genetic variations within or around *TaSNAC* genes associated with specific drought tolerance traits, an association analysis was conducted using the genotypic SNP data from the 39 *TaSNAC* genes and the drought tolerance phenotypic data from the 700 varieties. Three statistical models, including the general linear model (GLM), GLM + PCA and a mixed linear model (MLM), were applied to identify significant genotypic and phenotypic associations. Compared to GLM and GLM + PCA model, our results clearly showed that the MLM model significantly reduced the false‐positive associations (Figure S4).

**Table 1:** Association analysis of natural variations around *TaSNAC* genes with drought tolerance at seedling stage in the wheat diversity panel

Gene ID	Name	Chr.	Polymorphic	GLM	GLM + PCA	MLM
			Number*	(*P *< = 0.01)	(*P *< = 0.01)	(*P *< = 0.01)
TraesCS1A02G263700	TaSNAC1‐1A	1A	51	2	0	0
TraesCS1B02G274300	TaSNAC1‐1B	1B	14	2	0	0
TraesCS1D02G263800	TaSNAC1‐1D	1D	15	1	0	0
TraesCS3A02G406000	TaSNAC2‐3A	3A	42	3	1	0
TraesCS3B02G439600	TaSNAC2‐3B	3B	13	1	1	1
TraesCS3D02G401200	TaSNAC2‐3D	3D	33	5	2	0
TraesCS4A02G219700	TaSNAC3‐4A	4A	8	0	0	0
TraesCS4B02G098200	TaSNAC3‐4B	4B	22	0	0	0
TraesCS4D02G094400	TaSNAC3‐4D	4D	24	1	0	0
TraesCS3A02G339600	TaSNAC4‐3A	3A	15	0	0	0
TraesCS3B02G371200	TaSNAC4‐3B	3B	21	1	0	0
TraesCS3D02G333100	TaSNAC4‐3D	3D	12	0	0	0
TraesCS5A02G468300	TaSNAC5‐5A	5A	14	1	0	0
TraesCS5B02G480900	TaSNAC5‐5B	5B	20	0	0	0
TraesCS5D02G481200	TaSNAC5‐5D	5D	12	2	0	0
TraesCS6A02G057400	TaSNAC6‐6A	6A	36	1	1	0
TraesCS6B02G075200	TaSNAC6‐6B	6B	12	0	0	0
TraesCS6D02G059300	TaSNAC6‐6D	6D	17	0	0	0
TraesCS2A02G201800	TaSNAC7‐2A	2A	4	1	0	0
TraesCS2B02G228900	TaSNAC7‐2B	2B	6	0	0	0
TraesCS2D02G214100	TaSNAC7‐2D	2D	9	1	0	0
TraesCS6A02G108300	TaSNAC8‐6A	6A	57	41	15	10
TraesCS6B02G207500	TaSNAC8‐6B	6B	12	1	0	0
TraesCS6D02G096300	TaSNAC8‐6D	6D	19	5	0	0
TraesCS3A02G078400	TaSNAC9‐3A	3A	21	1	0	0
TraesCS3B02G093300	TaSNAC9‐3B	3B	49	2	1	0
TraesCS3D02G078900	TaSNAC9‐3D	3D	26	3	1	1
TraesCS5A02G143200	TaSNAC10‐5A	5A	3	0	0	0
TraesCS5B02G142100	TaSNAC10‐5B	5B	12	1	0	0
TraesCS5D02G148800	TaSNAC10‐5D	5D	17	2	0	0
TraesCS4A02G131000	TaSNAC11‐4A	4A	3	0	0	0
TraesCS4B02G173600	TaSNAC11‐4B	4B	3	2	0	0
TraesCS4D02G175700	TaSNAC11‐4D	4D	9	0	0	0
TraesCS2A02G101900	TaSNAC12‐2A	2A	32	2	1	1
TraesCS2B02G119000	TaSNAC12‐2B	2B	9	0	0	0
TraesCS2D02G101300	TaSNAC12‐2D	2D	19	1	0	0
TraesCS2A02G102000	TaSNAC13‐2A	2A	31	2	1	1
TraesCS2B02G119100	TaSNAC13‐2B	2B	10	0	0	0
TraesCS2D02G101400	TaSNAC13‐2D	2D	21	2	0	0

* MAF (minor allele frequency)> = 0.05.

The analysis detected 10 SNP markers around *TaSNAC8‐6A* significantly associated with drought tolerance in this wheat panel under the MLM model (*P* < 0.01), including the most significantly associated SNP marker out of the total 753 SNPs detected around all *TaSNAC* genes (*P* = 3.29 × 10^‐4^) (Table 1; Figure S5a). We next analysed the 10 predicted genes within the 200‐kb interval centred on *TaSNAC8‐6A* (Figure S5b). We selected two lines each from the drought‐tolerant (Pubing202) and drought‐sensitive (Yangmai13) varieties to measure expression of these 10 genes under drought conditions by qRT‐PCR (Figure S5c). Of the 10 genes, four showed slight up‐regulation under drought stress in either the drought‐sensitive or drought‐tolerant varieties. Only *TaSNAC8‐6A* (*TraesCS6A02G108300*) showed roughly twenty‐fold higher expression under drought stress in both the drought‐sensitive and drought‐tolerant varieties (Figure S5c). Thus, *TaSNAC8‐6A* was revealed to be a potentially important candidate gene significantly associated with drought tolerance in this wheat natural variation panel.

### Genetic variation in *TaSNAC8‐6A* contributes to wheat drought tolerance

**Figure 3:**
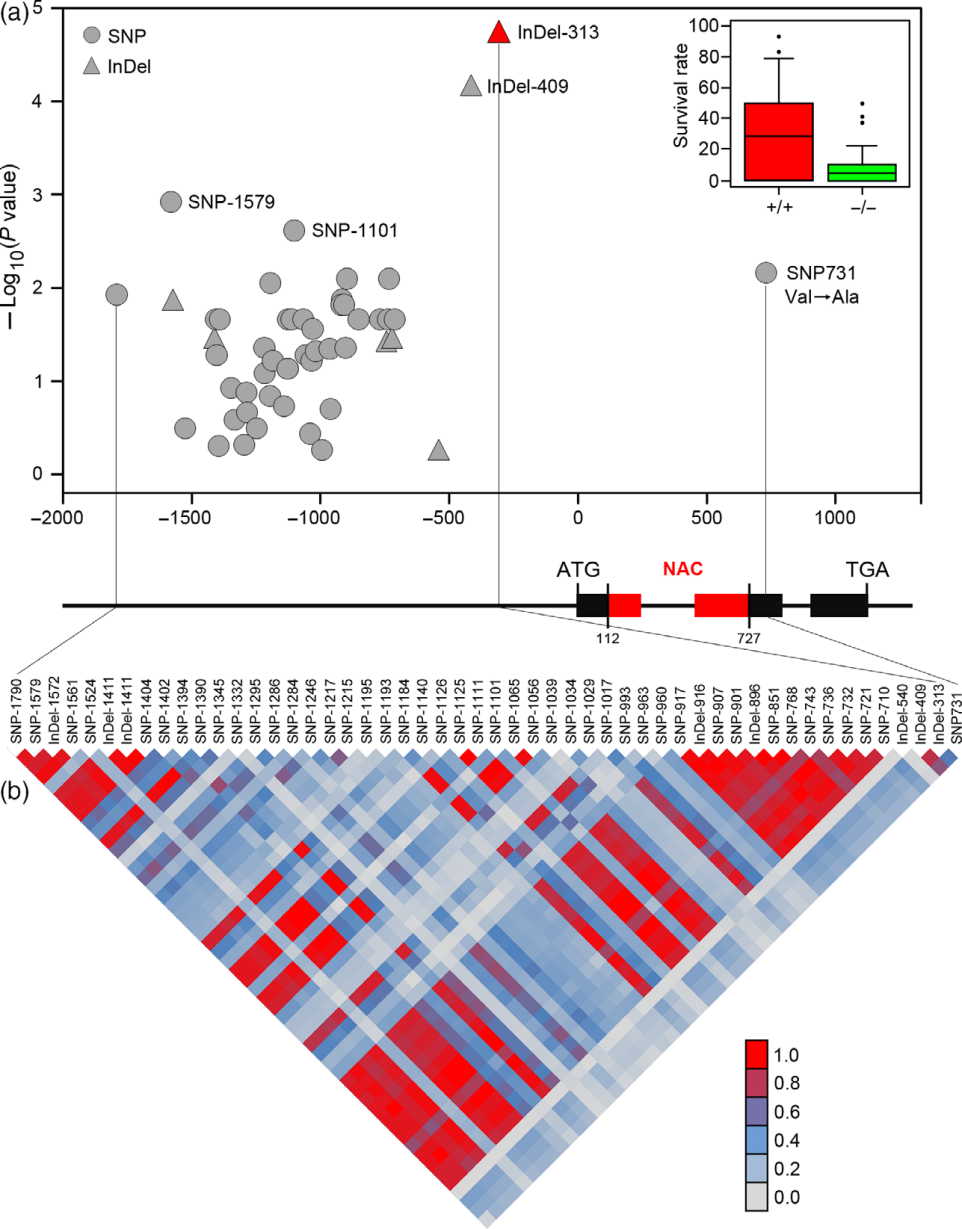
Association analysis of genetic variation in *TaSNAC8‐6A* with wheat drought tolerance. (a) Association of SNPs/InDels from *TaSNAC8‐6A* with drought tolerance phenotypes. 51 SNPs/InDels were used in the association analysis, and InDel‐313 was most significantly associated with drought tolerance compared to other SNPs/InDels. The *P* value is shown on a ‐log_10_ scale. (b) The pattern of pairwise LD of DNA polymorphisms in the *TaSNAC8‐6A* gene. InDel‐313 shows strong linkage disequilibrium (R^2^> 0.8) with InDel‐409.

To accurately identify genetic variations, *TaSNAC8‐6A* was resequenced in 109 randomly selected variation panel accessions. The sequenced region encompassed a 3310‐bp genomic DNA fragment corresponding to the full‐length *TaSNAC8‐6A* locus, including its promoter, exons, introns and both the 5'‐ and 3'‐UTRs. In total, 43 SNPs and eight insertion/deletions (InDels) were identified (Table S3). An MLM‐based association analysis was applied between all SNPs/InDels and the previously assigned drought tolerance phenotypes for each accession. Two newly identified genetic polymorphisms in the *TaSNAC8‐6A* promoter were highly associated with drought tolerance (*P* < 1.0 × 10^−3^), and specifically, InDel‐313 showed the strongest association (*P* = 1.67 × 10^−5^). Additionally, one nonsynonymous SNP (SNP731; *P* = 6.92 × 10^−3^) was identified in the coding region (Figure 3a; Table S3). The pairwise LD of these SNP markers indicated that InDel‐313 was in high LD with InDel‐409, but in low LD with other DNA polymorphisms (Figure 3b).

As the most significant variants were located in the promoter region, we then analysed the expression of *TaSNAC8‐6A* under well‐watered (relative leaf water content (RLWC) = 90%), moderate drought (RLWC = 72%) and severe drought (RLWC = 58%) conditions in 40 wheat varieties, including 18 drought‐tolerant and 22 drought‐sensitive varieties, in order to identify stress‐related regulatory differences in *TaSNAC8‐6A* expression. Under well‐watered or severe drought conditions, there were no significant differences in *TaSNAC8‐6A* transcription between drought‐tolerant and drought‐sensitive varieties (Figure S6a,c). However, under moderate drought, expression of *TaSNAC8‐6A* was significantly higher in tolerant varieties than in sensitive ones (Figure S6b), suggesting that up‐regulation of *TaSNAC8‐6A* is closely associated with higher tolerance to drought in wheat. Furthermore, the 18 drought‐tolerant varieties all carry an insertion allele (*TaSNAC8‐6A* ^In‐313^), while the 22 drought‐sensitive varieties possess a deletion allele (*TaSNAC8‐6A* ^Del‐313^). Taken together with expression analysis, these data indicate that *TaSNAC8‐6A* regulation is closely associated with the DNA polymorphisms InDel‐313.

To further compare the effects of the *TaSNAC8‐6A* ^In‐313^ and *TaSNAC8‐6A* ^Del‐313^ alleles on drought tolerance in wheat seedlings, we constructed two bi‐parental F_2_ populations by crossing drought‐tolerant variety Pubing202 with two drought‐sensitive varieties, Yangmai13 and GLUYAS EARLY. In these crosses, both Yangmai13 and GLUYAS EARLY have the *TaSNAC8‐6A* ^Del‐313^ alleles, while Pubing202 carries the *TaSNAC8‐6A* ^In‐313^ allele (Figure S6d). qRT‐PCR expression analysis showed that *TaSNAC8‐6A* expression was higher in Pubing202, regardless of water availability, compared to Yangmai13 and GLUYAS EARLY (Figure S6e). To more quantitatively determine the phenotypic contributions of the *TaSNAC8‐6A* ^Del‐313^ and *TaSNAC8‐6A* ^In‐313^ alleles to drought tolerance, the two F_2_ segregation populations were treated with drought, and the plant survival rates after stress and re‐watering were calculated. In the population derived from Yangmai13 × Pubing202, the survival rate of *TaSNAC8‐6A* ^Del‐313^ homozygous plants was ~ 36%, lower than the survival rate of *TaSNAC8‐6A* ^In‐313^ homozygous plants (58%). Similarly, in the population derived from GLUYAS EARLY × Pubing202, the survival rate was ~ 33% for plants with a homozygous *TaSNAC8‐6A* ^Del‐313^ allele, also lower than the survival rate of *TaSNAC8‐6A* ^In‐313^ homozygous plants (67%) (Figure S6f). Collectively, these data further support the premise that variation in *TaSNAC8‐6A* contributes to the drought tolerant phenotype. In particular, the strong performance of plants carrying *TaSNAC8‐6A* ^In‐313^ in drought conditions suggests that this allele represents a promising genetic resource for improvement of drought tolerance in more susceptible, high‐yield wheat lines.

### *TaSNAC8‐6A* ^In‐313^ allele increases drought‐activated transcriptional response to ABA signalling

**Figure 4:**
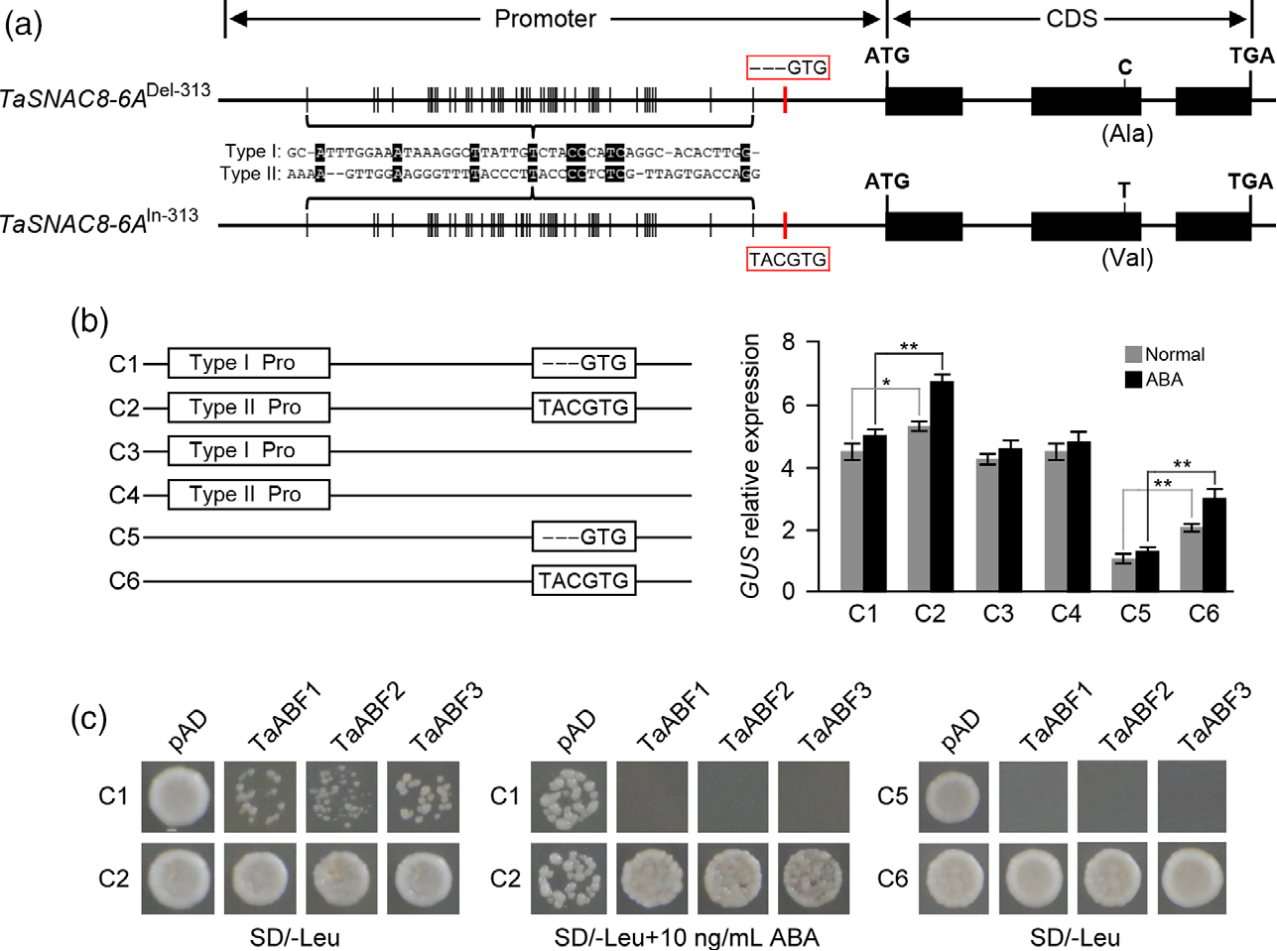
Identification of *TaSNAC8‐6A* as a direct target of TaABFs. (a) Diagram of the ABRE sequence motif (TACGTG) in the *TaSNAC8‐6A* ^Del‐313^ (top) and *TaSNAC8‐6A* ^In‐313^ (bottom) genotypes. (b) Comparison of the effects on *GUS* reporter gene expression of two promoter sequence variants (with or without ABRE motif). Error bars, SD; statistical significance was determined by a two‐sided *t*‐test, *P < 0.05, **P < 0.01. (c) Yeast‐one hybrid assay of TaABFs binding to the *TaSNAC8‐6A* promoter fragments. The bait vectors containing full‐length (C1 and C2) or truncated (C5 and C6) promoters and the prey vector containing *TaABFs* were introduced into yeast strain Y187. Yeast cells were grown on SD‐Leu media with or without ABA.

Sequence analysis of *TaSNAC8‐6A* InDel‐313 revealed that the 3‐bp insertion/deletion (TAC) was located in an ABRE motif (TACGTG) (Figure 4a). To verify whether the location of InDel‐313 in the promoter region affected transcriptional levels of *TaSNAC8‐6A*, we inserted promoter fragments from both *TaSNAC8‐6A* ^In‐313^ and *TaSNAC8‐6A* ^Del‐313^ upstream of a *GUS* (*β*‐glucuronidase) to compare their transcriptional activation of the reporter in the presence and absence of ABA (Figure 4a,b). We found that regardless of whether plants were exposed or not to ABA, *GUS* expression driven by the *TaSNAC8‐6A* ^In‐313^ promoter (C2; tolerant) was much higher than that driven by the *TaSNAC8‐6A* ^Del‐313^ promoter (C1; sensitive). *GUS* expression was comparable to that of the C1 fragment when driven by the truncated promoter variants *TaSNAC8‐6A* ^In‐313^ (C4) and *TaSNAC8‐6A* ^Del‐313^ (C3), which lacked InDel‐313 and the ABRE motif altogether. Furthermore, *GUS* expression driven by a truncated *TaSNAC8‐6A* ^In‐313^ promoter variant carrying only the In‐313/ABRE motif region (C6 construct) was significantly higher than that driven by the truncated *TaSNAC8‐6A* ^Del‐313^ promoter variant carrying only the Del‐313/ABRE motif (C5 construct). In addition, exposure to 10 µm ABA significantly increased *GUS* expression driven either by the full length (C2) and truncated (C6) *TaSNAC8‐6A* ^In‐313^ promoter fragments compared to their expression in the absence of ABA, though the C6 variant was considerably lower than the full length In‐313 C2 construct (Figure 4b). These results showed that InDel‐313 is a causal genetic element for *TaSNAC8‐6A* up‐regulation, and the insertion at this indel site increased *TaSNAC8‐6A* expression.

In plants, AREB/ABF TFs are reported to directly target a core ABRE motif present in target gene promoters (Fujita *et al.*, 2013). In order to identify the candidate AREB/ABF proteins that bind the ABRE motif in the *TaSNAC8‐6A* promoter, we searched the wheat genome database using *Arabidopsis* AREB/ABFs as queries. We thus identified nine ABFs in wheat and designated them as TaABF1, TaABF2 and TaABF3 followed by chromosome locations. Phylogenetic analysis showed that TaABF1 was closely related to *Arabidopsis* ABF1/3/4, while TaABF2 and TaABF3 were closely related to ABF2 (Figure S7a). qRT‐PCR analysis revealed that all *TaABFs* were predominantly expressed in seedling leaves and were up‐regulated in response to drought in leaf tissue (Figure S7b,c). We then conducted yeast one‐hybrid assays, which indicated that TaABF1, TaABF2 and TaABF3 are candidate *TaSNAC8‐6A* ABRE‐binding proteins (Figure 4c). Furthermore, yeast cells carrying either the *TaSNAC8‐6A* ^Del‐313^ (C1) or truncated *TaSNAC8‐6A* ^Del‐313^, consisting only of Del‐313 (C5), promoter fragments grew slowly and with less binding activity than those transformed with the *TaSNAC8‐6A* ^In‐313^ promoter fragment (C2) or truncated *TaSNAC8‐6A* ^In‐313^, carrying only In‐313 (C6). These differences increased with ABA concentration (Figure 4c). Taken together, these data clearly indicate that TaABFs can directly bind with stronger affinity to the ABRE motif in the *TaSNAC8‐6A* ^In‐313^ promoter than to that of *TaSNAC8‐6A* ^Del‐313^ alleles to increase the *TaSNAC8‐6A* expression and therefore enhance wheat drought tolerance.

### Overexpression of *TaSNAC8‐6A* confers seedling drought tolerance

**Figure 5:**
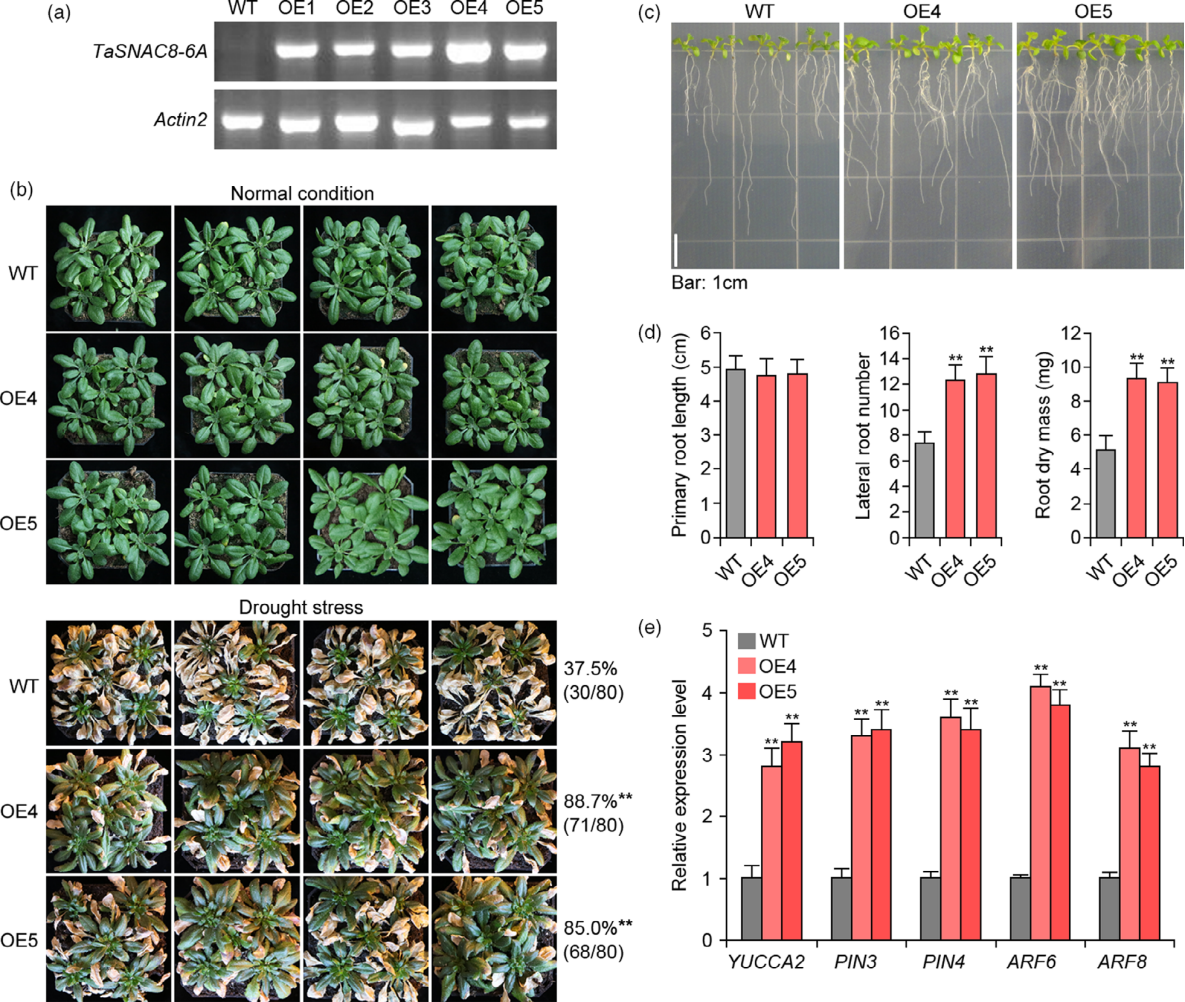
Drought tolerance of *35S:TaSNAC8‐6A* transgenic *Arabidopsis* plants. (a) RT‐PCR analysis of *TaSNAC8‐6A* transcription levels in the five *35S:TaSNAC8‐6A* transgenic overexpression lines. (b) Assessment of drought tolerance in WT and *35S:TaSNAC8‐6A* transgenic plants. (c) Phenotypic comparisons of lateral‐root development between WT and *35S:TaSNAC8‐6A* transgenic plants. (d) Statistical analysis of primary root length, lateral root number and root dry mass of WT and *35S:TaSNAC8‐6A* transgenic plants. (e) Relative expression levels of auxin‐related genes in WT and *35S:TaSNAC8‐6A* transgenic *Arabidopsis* roots under well‐watered conditions.

To better understand *TaSNAC8‐6A* function in drought tolerance, we generated transgenic *Arabidopsis* expressing *TaSNAC8‐6A* coding sequence from wheat *cv.* Chinese Spring. Two independent lines exhibiting high *TaSNAC8‐6A* expression were selected for characterization of their stress response to drought (Figure 5a). We compared drought tolerance between transgenic and vector‐transformed (WT) plants and found that transgenic *Arabidopsis* displayed significantly more robust drought tolerance, without inducing dramatic morphological changes under normal growth conditions. When WT survival was ~37%, ~85% of transgenic *Arabidopsis* plants survived in parallel water‐withholding experiments (Figure 5b). The transgenic plants also developed more lateral roots and greater root dry mass with no difference in primary root length (Figure 5c,d). Previous reports revealed that NAC TFs selectively bind to either T[TAG][GA]CGT[GA][TCA][TAG] or [ACG][CA]GT[AG]N{5,6} [CT]AC[AG] consensus sequences (Matallana‐Ramirez *et al.*, 2013; Olsen *et al.*, 2005). Analysis of promoter regions or 5'‐UTRs of auxin‐related genes *YUCCA2*, *PIN3*, *PIN4*, *ARF6* and *ARF8* confirmed the presence of one or both NAC‐binding sites, suggesting these genes can potentially serve as *TaSNAC8‐6A* regulatory targets. Expression of these auxin‐related genes was elevated in transgenic *Arabidopsis* compared to WT, which likely contributed to their enhanced root system development (Figure 5e).

**Figure 6:**
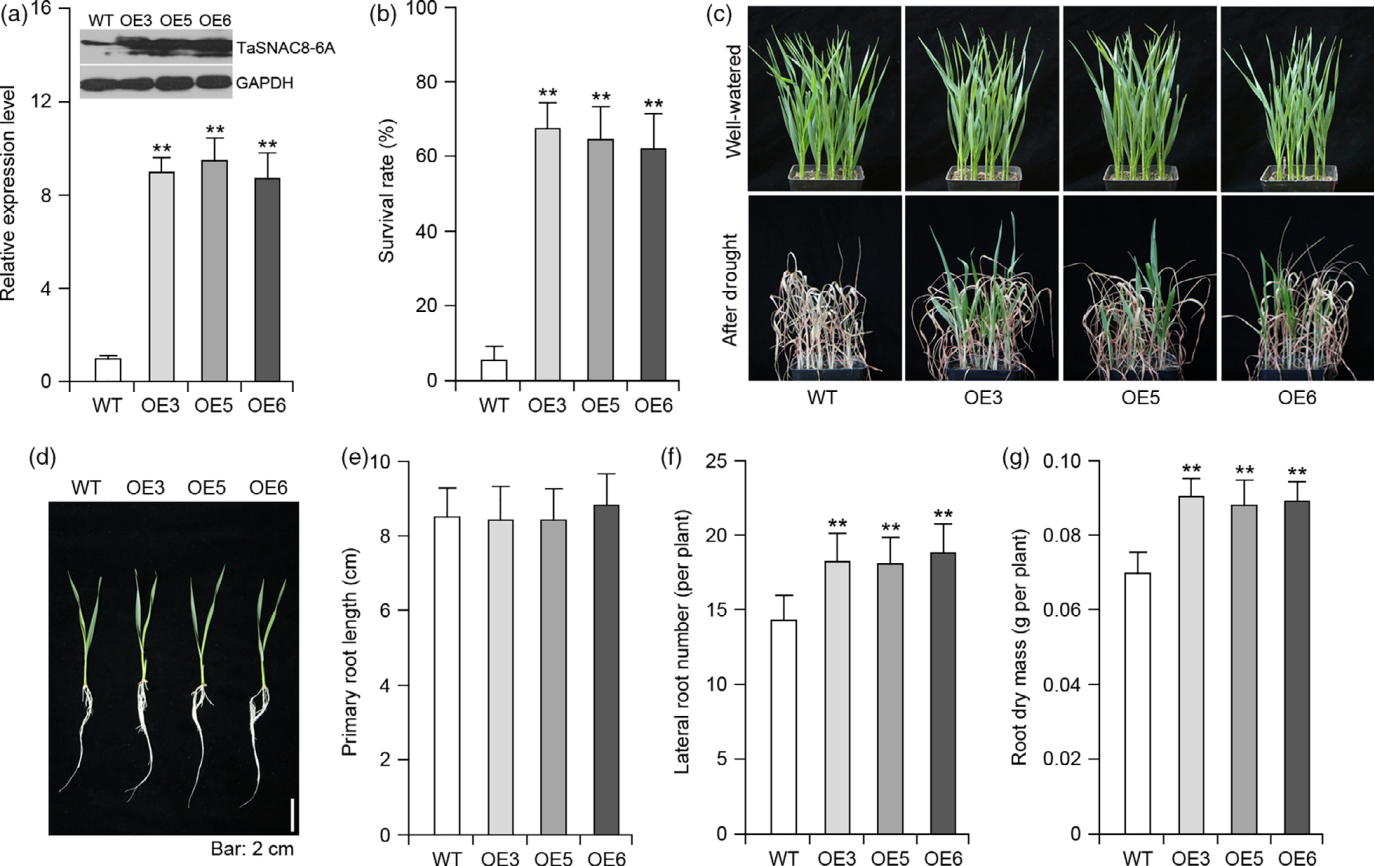
Drought tolerance of *Ubi:TaSNAC8‐6A* transgenic wheat. (a) Protein‐blot analysis of TaSNAC8‐6A (top) and relative expression levels of *TaSNAC8‐6A* (bottom) in WT and three independent *Ubi:TaSNAC8‐6A* transgenic lines grown under well‐watered conditions. (b) Statistical analysis of the survival rate of WT and *Ubi:TaSNAC8‐6A* transgenic lines exposed to drought stress. (c) Assessment of drought tolerance in WT and *Ubi:TaSNAC8‐6A* transgenic lines. (d) Phenotypic comparison of lateral‐root development between WT and *Ubi:TaSNAC8‐6A* transgenic lines. (e‐g) Statistical comparisons of primary root length (e), lateral root numbers (f) and root dry mass (g) for WT and *Ubi:TaSNAC8‐6A* transgenic lines under well‐watered conditions.

In order to explore the effects of constitutively *TaSNAC8‐6A* expression in wheat, we overexpressed this gene in the wheat *cv*. Fielder background and subsequently selected three independent overexpression lines for further testing. Protein blot analyses demonstrated that *TaSNAC8‐6A* was properly expressed, translated and functional in transgenic wheat (Figure 6a). We then compared drought tolerance through side‐by‐side planting of transgenic and WT siblings and consistently observed two‐ to threefold increases in survival rate for transgenic lines over WT under water‐deficit conditions (Figure 6b,c)*.* Root system comparisons between overexpression lines and WT recapitulated findings in *Arabidopsis* that primary root lengths were comparable between transgenic and WT, while lateral root number and root dry mass were substantially increased in transgenic wheat compared to WT (Figure 6d‐g).

To determine whether *TaSNAC8‐6A* overexpression affected yield in addition to improving drought tolerance, we preliminary evaluated production under well‐watered and water‐limited conditions in the greenhouse. Under well‐watered conditions, phenotype of transgenic plants was almost identical to WT (Figure S8a), and similar to observations of seedling roots, transgenic lines produced more lateral roots than WT (Figure S8b). Furthermore, water use efficiency (WUE) in the transgenic lines was significantly elevated over that of WT under drought stress, but not significantly different under well‐watered conditions (Figure S8c). Moreover, yield‐related traits of transgenic plants were also similar to that of WT (Figure S8d). However, under water‐limited conditions, the transgenic lines exhibited slightly later flowering, wider leaves and higher grain yield than WT (Figure S8e). In the present study, yield‐related traits were investigated mainly in the greenhouse. Whether *TaSNAC8‐6A* can significantly contribute to wheat yield in the field requires further detailed field‐based investigation. Collectively, these data suggest that increased *TaSNAC8‐6A* expression may improve auxin‐related lateral root development and WUE, which further contribute to the enhanced drought tolerant phenotype.

### *TaSNAC8‐6A* overexpression induces global changes to gene expression particularly in auxin‐ and drought‐responsive signalling pathways

To better understand the regulatory network through which *TaSNAC8‐6A* mediates a strongly tolerant phenotypic response to drought, we compared the transcriptomes of WT and transgenic OE3 and OE5 lines under both well‐watered and water‐deficit conditions. Under well‐watered conditions, a total of 1451 and 554 genes (adjusted *P* < 0.01, fold change > 2 or < 0.5) were up‐regulated and down‐regulated, respectively, between transgenic lines and WT (Figure S9a,b; Table S4). Gene ontology (GO) analysis revealed up‐regulated genes in ‘chitin metabolism’, ‘secondary metabolism’, ‘response to abiotic stress’, ‘response to drought’ and ‘response to auxin’ biological pathways. In contrast, down‐regulated genes were found in ‘homeostatic process’, ‘energy quenching’ and ‘response to oxidative stress’ biological pathways (Figure S9c). These results support the qRT‐PCR relative expression data indicating that *TaSNAC8‐6A* up‐regulates stress‐ and auxin‐related genes.

**Figure 7:**
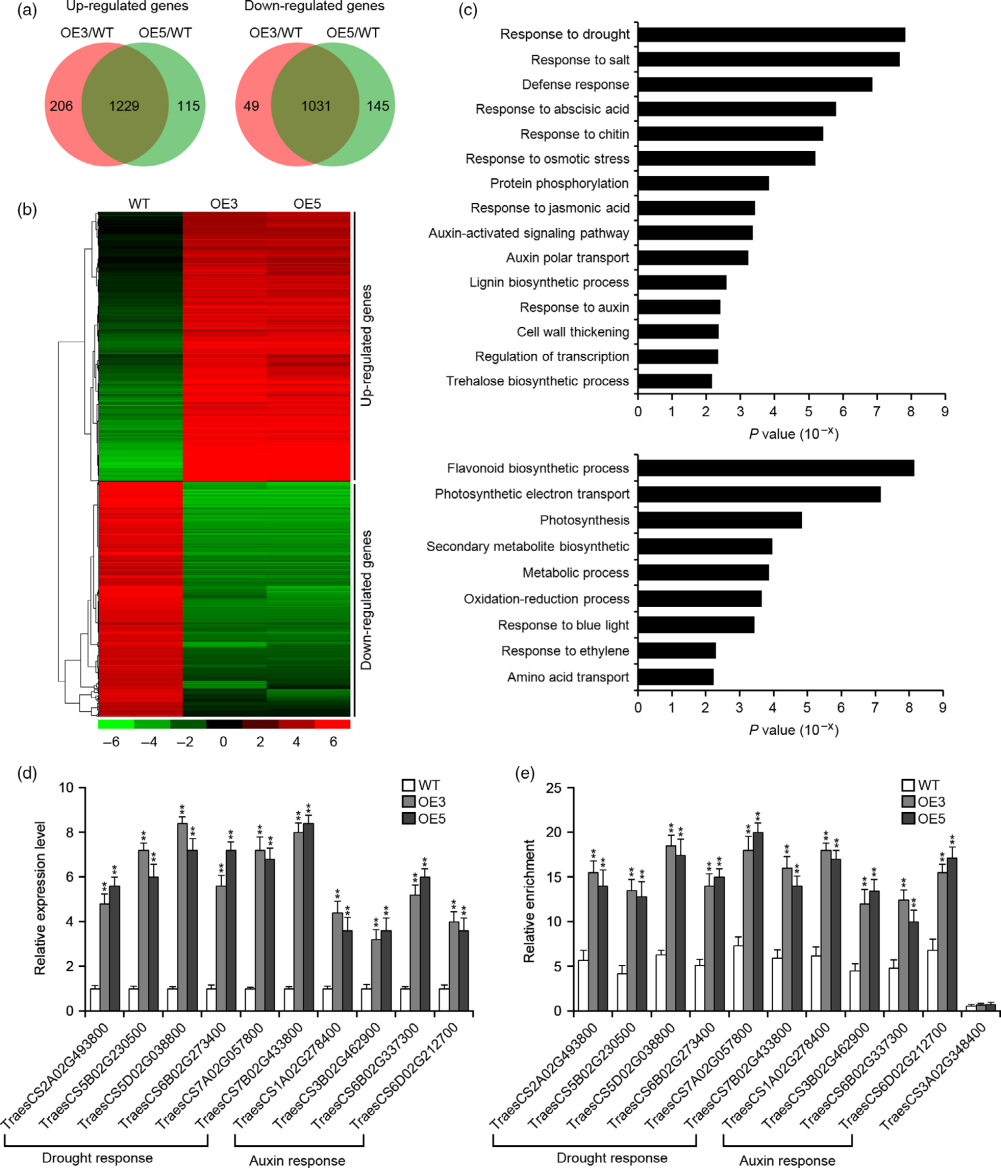
Transcriptomic analysis of *Ubi:TaSNAC8‐6A* transgenic wheat. (a) Venn diagrams of up‐ or down‐regulated genes in transgenic OE3 and OE5 lines. (b) Hierarchical clustering of differentially expressed genes in the transgenic lines relative to WT plants. The indicated scale is the log_2_ value of the normalized level of gene expression. (c) Gene ontology of biological pathways enriched in the transgenic lines based on the up‐ or down‐regulated genes. (d) qRT‐PCR verification of increased expression of selected genes involved in water and auxin response in the transgenic and WT plants. (e) ChIP‐qPCR analysis of selected genes involved in water and auxin response in the transgenic and WT plants. Relative enrichment of fragments was calculated by comparing samples immunoprecipitated with antibodies to TaSNAC8‐6A and GAPDH. The non‐target gene *TraesCS3A02G348400* serves as a control.

Under drought stress, 1229 and 1031 genes (adjusted *P* < 0.01, fold change > 2 or < 0.5) were at least twofold up‐ and down‐regulated, respectively, in the transgenic lines compared to WT (Figure 7a,b; Table S5). GO analysis showed that up‐regulated genes found in stimulus‐related pathways, including ‘response to drought’, ‘response to abscisic acid (ABA)’ and ‘response to osmotic stress’. Interestingly, ‘auxin‐activated signalling pathway’ and ‘auxin polar transport’ biological pathways had genes up‐regulated under drought conditions, whereas ‘flavonoid biosynthetic’, ‘secondary metabolite’, ‘photosynthesis’ and ‘oxidation‐reduction process’ pathways contained down‐regulated genes (Figure 7c). Furthermore, more genes categorized as ‘drought response’, ‘ABA response’ and ‘auxin response’ were up‐regulated in drought‐stressed samples than in well‐watered samples (Figure 7c; Table S6). We hypothesized that these transcriptomic changes likely enhance root development in transgenic wheat to improve WUE during drought by increasing the roots’ capacity to absorb soil moisture.

An increase in expression was verified for several drought‐inducible and auxin‐related genes in transgenic lines, including the wheat homologs of *Arabidopsis OST1* (*TraesCS2A02G493800*), *RZF1* (*TraesCS5B02G230500*), *NCED3* (*TraesCS5D02G038800*), *COR47* (*TraesCS6B02G273400*), *AVP1* (*TraesCS7B02G433800*), *DREB1A* (*TraesCS7A02G057800*), *AUX1* (*TraesCS1A02G278400*), *PIN5* (*TraesCS3B02G462900*), *PIN3* (*TraesCS6B02G337300*) and *ARF16* (*TraesCS6D02G212700*) (Figure 7d). All of these genes contain the NAC recognition core sequence (CACG) in their promoters (Table S6), suggesting that they are strong potential target genes of TaSNAC8‐6A. We next used ChIP‐qPCR to detect the binding sites near the NAC recognition core sequence in the promoters of these candidate target genes. The results revealed that TaSNAC8‐6A can directly bind to the promoters of these up‐regulated genes (Figure 7e), thus confirming their participation in TaSNAC8‐6A regulatory networks during drought stress response.

## Discussion

### Regulatory variation in *TaSNAC8‐6A* correlates to wheat drought tolerance

Although many genes necessary for drought adaptation have been cloned (Hu and Xiong, 2014; Zhu, 2016), the contribution by natural sequence variation to phenotypic variations remains largely unknown. Previous research has shown that genetic changes in *cis* regulatory regions can drive advances in plant evolution and breeding, often through subtle phenotypic changes induced by modification of the timing, pattern, or level of gene expression (Meyer and Purugganan, 2013; Wittkopp and Kalay, 2011). Our analysis revealed that the promoter InDel‐313 polymorphism is highly associated with *TaSNAC8‐6A* expression. Specifically, varieties with *TaSNAC8‐6A* ^Del‐313^ show lower expression of *TaSNAC8‐6A* under moderate drought stress and tend to be drought sensitive, while varieties carrying *TaSNAC8‐6A* ^In‐313^ show higher expression and exhibit higher drought tolerance (Figure S6b). The main reason is that the *TaSNAC8‐6A* ^Del‐313^ allele lacks an ABRE motif and thus cannot bind with TaABFs, further lowering the expression of *TaSNAC8‐6A* (Figure 4). In addition, in the F_2_ segregation populations, *TaSNAC8‐6A* ^In‐313^ homozygous plants showed higher tolerance to drought stress, while plants carrying *TaSNAC8‐6A* ^Del‐313^ showed increased susceptibility to drought stress (Figure S6d‐f). We therefore propose that the *TaSNAC8‐6A* ^In‐313^ allele is favourable for adoption by breeding programmes seeking to improve drought tolerance in wheat germplasm.

### *TaSNAC8‐6A* plays a positive role in wheat drought tolerance

NAC TFs involved in regulating drought tolerance are promising targets in breeding for drought tolerant varieties (Hu and Xiong, 2014; Nakashima *et al.*, 2012). *TaSNAC8‐6A* expression was positively correlated with seedling drought tolerance under moderate drought stress, with higher expression observed among drought‐tolerant accessions across a panel of 40 wheat varieties (Figure S6b). When the *TaSNAC8‐6A* was overexpressed in *Arabidopsis* and wheat, the drought tolerance of transgenic plants was significantly improved (Figure 5a,b; Figure 6a‐c). These data suggest a positive regulatory role for *TaSNAC8‐6A* in adaptation to drought stress in wheat.

We also investigated the yield of transgenic *TaSNAC8‐6A* overexpression wheat lines and found that grain yields of overexpression plants were slightly higher under water‐limited conditions, while under well‐watered conditions, the yields were almost identical compared to WT (Figure S8d,e). Importantly, under well‐watered conditions, both transgenic lines exhibited more extensive root systems than WT (Figure S8b), which could plausibly contribute to the observed yield gain. In agreement with this finding, similar effects on yield have also been observed in transgenic rice overexpressing *OsNAC5*, *OsNAC6* and *OsNAC9* (Jeong *et al.*, 2013; Lee *et al.*, 2017; Redillas *et al.*, 2012). However, when *OsNAC5*, *OsNAC6* and *OsNAC9* were overexpressed in rice driven by a root‐specific (RCc3) promoter, the grain yields were significantly increased under both well‐watered and water‐limited conditions (Jeong *et al.*, 2013; Lee *et al.*, 2017; Redillas *et al.*, 2012). Future investigation would incorporate tissue‐specific promoters, such as RCc3, for optimization of *TaSNAC8‐6A* overexpression for wheat production under field conditions.

### *TaSNAC8‐6A* is involved in the regulation of wheat root development

Environmental disturbances have been tightly correlated with changes in root architecture, and to adapt to stress, plants can modulate the growth of primary, lateral or adventitious roots as well as root hair length and distribution (Malamy, 2005). The plant growth hormone auxin has been established as an influence on development of primary root length, number of lateral roots and gravitropism (Himanen *et al.*, 2002; Zhao, 2010). However, little is yet known about which genes in the auxin signalling pathway may be directly targeted by NAC transcription factors. *TaSNAC8‐6A* overexpression in both *Arabidopsis* and wheat enhanced their drought tolerance and improved root system development (Figure 5; Figure 6), which was also observed in transgenic *Arabidopsis* overexpressing *AtNAC2* (He *et al.*, 2005) and transgenic rice overexpressing *OsNAC6* (Lee *et al.*, 2017).

This study also revealed the presence of NAC binding sites in the promoters of the auxin‐related genes *YUCCA2*, *PIN3*, *PIN4*, *ARF6* and *ARF8.* Relative expression analysis showed that these genes were transcriptionally elevated in transgenic *Arabidopsis* plants, suggesting that they may be targets of *TaSNAC8‐6A* (Figure 5e). While in *TaSNAC8‐6A* transgenic wheat lines, root transcriptome analysis revealed significant up‐regulation of several auxin transporter genes and the auxin polar transport pathway were highly enriched (Figure 7c; Figure S9c; Table S6), in agreement with the known mechanisms of auxin translocation (Lavenus *et al.*, 2013; Matthes *et al.*, 2019). Our data also revealed similar up‐regulation of several wheat homologs of canonical auxin signal transduction genes, such as ARFs, EINs and Aux/IAA (Figure 7c; Figure S9c; Table S6). The increased expression of these auxin signalling and transport genes caused by *TaSNAC8‐6A* overexpression suggests a likely mechanism for enhanced root development that benefits plants during drought‐induced stress.

**Figure 8:**
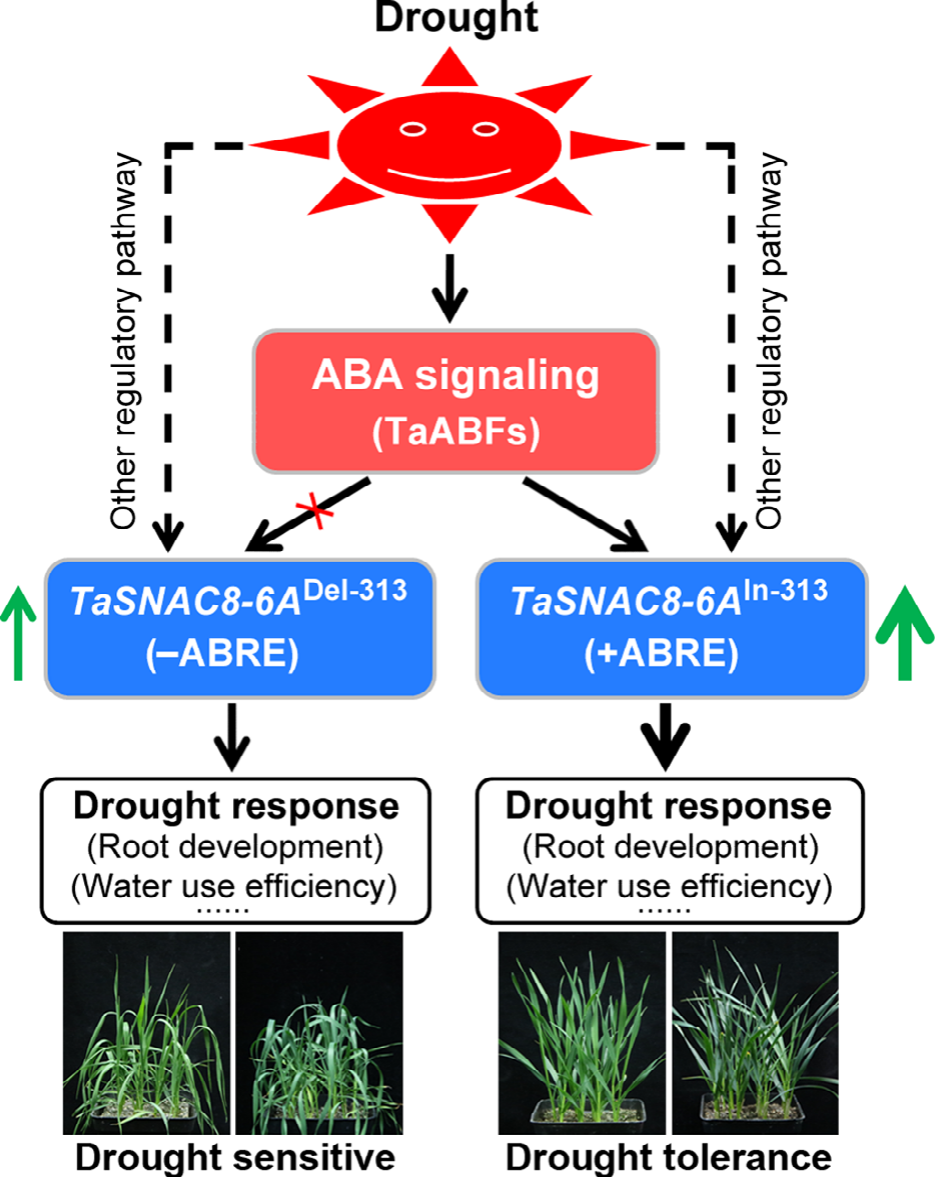
A proposed working model of InDel‐313. The model illustrates the role of InDel‐313 as a molecular switch in ABA signalling in regulation of *TaSNAC8‐6A* expression, and further in wheat drought tolerance.

Based on our results, we proposed a model of how InDel‐313 of *TaSNAC8‐6A* functions in drought response (Figure 8). Accordingly, drought stress activates *TaSNAC8‐6A* expression through ABA signalling or other regulatory pathways. In the ABA signalling pathway, drought could induce *TaABFs* expression, which activates *TaSNAC8‐6A* depending on the ABRE element in its promoter. The *TaSNAC8‐6A* ^In‐313^ allele has an ABRE element, which can bind with TaABFs and further enhance the expression of *TaSNAC8‐6A*. The overproduced *TaSNAC8‐6A* more strongly promotes drought response and further causes improved drought tolerance of wheat varieties. Thus, the natural variation of InDel‐313 most likely functions as a molecular switch in ABA signalling to regulate *TaSNAC8‐6A* expression and further plant drought tolerance.

## Experimental procedures

### Expression analysis of *TaSNAC* genes

Seeds of wheat *cv*. Chinese Spring were surface‐sterilized in 1.0 % (v/v) Topsin‐M (Rotam Crop Sciences Ltd., Taiwan, China) for 10 min, washed in deionized water and germinated on wet filter paper (25 °C; 3 days). Germinated seeds were transplanted to hydroponic nutrient solution. For drought treatment, the three‐leaf‐stage seedlings were dehydrated at 20 °C, 40–60 % relative humidity. Leaves and roots were collected at 0, 1, 3, 6, 12 and 24 h after dehydration. Total RNA was isolated using TRIZOL reagent (Invitrogen, Carlsbad, CA), and subsequently, all samples were treated with DNase I (Takara, Dalian, China) and RNA concentration was determined by Nanodrop 1000 (Thermo Scientific, Shanghai, China). cDNA was prepared using M‐MLV Reverse Transcriptase, and qRT‐PCR used SYBR Premix Ex Taq kit (Takara) on a Step One System (Applied Biosystems, Shanghai, China). The 2^−∆∆Ct^ method was used to calculate relative expression using three biological replicates per sample. PCR conditions consisted of 95 °C for 10 min, 40 cycles at 95 °C for 15 s and 60 °C for 30 s.

### Yeast one‐hybrid transactivation activity assay

Thirteen *TaSNAC* genes were individually cloned into the pGBKT7 vector to evaluate protein transactivation activity in the AH109 yeast strain. All transformed cells were screened on synthetic defined (SD) medium plates without tryptophan (SD/‐T). The cell concentration of yeast transformants was adjusted to an OD_600_ of 0.1, and the yeast cells were drop‐inoculated onto SD/‐T plates, or SD without tryptophan and histidine (SD/‐T‐H), or without tryptophan, histidine and adenine (SD/‐T‐H‐A). The plates were incubated at 30 °C for 3‐6 days before photographing.

### Drought tolerance evaluation of the wheat association panel

700 wheat varieties were phenotyped for drought tolerance in the cultivation pool (9.0 × 1.0 × 0.2 m, length × width × depth) in which 0.6‐tons soil and 0.16‐tons vermiculite were uniformly mixed with 0.35‐tons water. The pool was divided into 700 plots and twelve replicate plants from each variety were grown together in a plot. Watering was withheld after seeds were planted and seedlings were grown in a greenhouse (16‐h light/8‐h dark light regimen, 16/14 °C and relative humidity of 40 %). Soil water content (SWC) was recorded beginning at the first symptoms of leaf‐wilting. Irrigation was applied to recover the surviving plants when clear differences in wilting were observed. Three days after rehydration, the survival rate (SR) of each genotype was recorded. Seedlings with viable green leaves were rated as survivors. Four independent, replicated experiments were conducted.

### Association analysis of *TaSNAC* genes

Association analysis was conducted on 700 wheat varieties with corresponding phenotypic drought tolerance data obtained in this study. Among 623 529 high‐quality wheat SNP markers with minor allele frequency (MAF) ≥ 0.05, 753 SNPs were found in the 400‐kb interval around all 39 *TaSNAC* genes. The GLM model, GLM model with principle components (GLM + PCA) and the MLM model were all tested for detection of SNPs significantly associated with drought tolerance using the TASSEL 5.0 program (Bradbury *et al.*, 2007). *TaSNAC8‐6A‐*based association mapping was performed on 109 representative wheat varieties. The *TaSNAC8‐6A* promoter (~2.0 kb), coding regions (including introns) and 5'‐ and 3'‐UTR sequences were amplified and sequenced. These sequences were assembled using DNAMAN and aligned using MEGA version 7. Nucleotide polymorphisms, including SNPs and InDels, were identified (MAF ≥ 0.05) among these genotypes and their association with the SR and pairwise LD were calculated with TASSEL 5.0.

### *Arabidopsis* transformation and drought tolerance assay

The *TaSNAC8‐6A* coding region in wheat *cv.* Chinese Spring was amplified and inserted at *Hind*III and *Sma*I sites into the pGreen vector (Qin *et al.*, 2008), driven by the *CaMV35S* promoter. The plasmid construct was transformed into *Agrobacterium tumefaciens* strain GV3101 containing the pSoup helper plasmid. *Arabidopsis thaliana* ecotype Col‐0 was then transformed using the floral dip method. Seedlings (T_3_) with single *TaSNAC8‐6A* insertions were identified for further analysis. For drought tolerance assay, seven‐day‐old plants were transferred into pots containing 130g soil/pot. At 30 days after germination, well‐watered plants were stressed by withholding water. Drought treatment lasted for 14 days, followed by a 6‐day recovery with watering prior to counting the surviving plants. Minimum 20 plants of each line were compared with WT, and four independent experiments were conducted. For analysis of lateral‐root development, seedlings were cultured on MS medium for 7 days and then transferred to fresh MS. Images were taken after 16 days and root dry mass was obtained from 30‐day‐old soil‐grown plants. After cleaning, total roots were dried at 70 °C for 24 h prior to measuring total root mass. Expression of auxin‐related genes was determined by qRT‐PCR from root samples of 16‐day‐old seedlings grown on MS.

### Wheat transformation and drought tolerance assay

The *TaSNAC8‐6A* CDS from wheat *cv.* Chinese Spring was inserted into the pCAMBIA3301 overexpression vector driven by an optimized wheat ubiquitin promoter. *Agrobacterium tumefaciens*‐mediated transformation introduced the plasmid into immature embryos of wild‐type bread wheat (*cv*. Fielder) following the protocol reported by Ishida (Ishida *et al.*, 2015). Transgenic‐positive and sibling transgenic‐negative (WT) plants in each generation were screened by PCR. The relative expression of *TaSNAC8‐6A* in transgenic plants was measured by qRT‐PCR, using protocols for RNA collection, cDNA synthesis and qRT‐PCR described above. Three independent T_3_ lines, OE3, OE5 and OE6, were planted side by side with WT in 2:1 soil:vermiculite. Three‐leaf soil‐grown seedlings underwent drought treatment for ~30 days, followed by a 3‐day recovery period with irrigation prior to counting surviving plants. At least 48 plants from each line were compared in each test, and three independent experiments were conducted. Lateral root development was measured using twenty‐day‐old hydroponic seedlings. Root material was then dried at 70 °C for 72 h to obtain dry mass.

### Evaluations of growth and yield components

Growth and yield trait evaluation of three independent T_3_ overexpression lines (OE3, OE5 and OE6) and sibling transgenic‐negative (WT control) plants, under well‐watered and mild‐drought conditions, were performed in plastic boxes (0.50 × 0.35 × 0.20 m, length × width × depth) in a 2:1 soil:vermiculite mixture. Each WT/transgenic line was grown in groups of four plants/row in two identical plastic boxes, one well‐watered and one with slowly increasing drought. Well‐watered plants were regularly irrigated until maturity. In drought‐subjected boxes, plants were regularly watered until the head sprouting period; then, irrigation was stopped. Plants showed mild wilting at the beginning of flowering. Soil water content was recorded every other day after the initiation of water withholding. Growth and yield characteristics for all lines and treatments were recorded, and statistical data were based on 8 seedlings per line. The experiment was repeated twice.

### RNA‐seq analysis of transgenic wheat

Twenty‐day‐old hydroponically cultivated *Ubi:TaSNAC8‐6A* transgenic and WT plants were used for root transcriptome analysis. Each total RNA sample was isolated using TRIzol reagent (Invitrogen) according to the manufacturer’s instructions and was evaluated for quality and quantity with an Agilent 2100 Bioanalyzer (Agilent Technologies, Palo alto, CA) and an Agilent RNA 6000 Nano Kit (Agilent Technologies). Library preparation was performed with a TruSeq RNA Sample Preparation version 2 guide (Illumina). Sequencing on an Illumina HiSeq 2500 system generated an average of 673 million paired‐end reads (2 × 101 nucleotides) per library. RNA‐seq data were analysed as described previously (Ramírez‐González *et al.*, 2018). Significant differential expression was defined as an absolute fold change > 2 and FDR < 0.01. GO categories matched with significantly up‐ and down‐regulated genes were identified using the GOseq R package (Young *et al.*, 2010).

### ChIP‐qPCR analysis

Twenty‐day‐old hydroponically cultivated *Ubi:TaSNAC8‐6A* transgenic wheat and WT plants were used for ChIP assays, performed according to previously described protocols (Bowler *et al.*, 2004). Seedlings were cross‐linked with 1 % formaldehyde under vacuum for 10 min and ground in liquid nitrogen prior to isolation of nuclei. DNA was sheared by sonication into 200‐ to 500‐bp fragments. Chromatin complexes were incubated with anti‐TaSNAC8‐6A polyclonal antibody, and immune complexes were pulled down using Protein A beads. The precipitated DNA was purified and dissolved in water for further analysis. A commercial polyclonal antibody to GAPDH (Santa Cruz Biotechnology, Santa Cruz, CA) was used as a negative control. All primers used in this research are listed in Table S7.

## Conflict of interest

The authors declare no conflict of interest.

## Author contributions

H.M. and Z.K. conceived the study and designed the experiments. H.M., S.L., Z.W., X.C., F.L., F.M. and N.C. performed the experiments. H.M. analysed the data. H.M. and Z.K. wrote the manuscript with contributions from all authors.

## Accession numbers

RNA‐seq raw reads have been deposited in the SRA as accession SRR9333957–SRR9333962.

## Supporting information


**Figure S1** Phylogenetic tree of stress responsive NAC proteins in wheat, rice, *B. distachyon*, maize, and Arabidopsis.

**Figure S2** Chromosome location and gene structure analysis of the TaSNAC genes.

**Figure S3** Phylogenetic tree, LD decay, and survival rates of the 700 wheat varieties.

**Figure S4** Quantile‐quantile (Q‐Q) plot for association of SNPs and drought tolerance.

**Figure S5** Identification and molecular characterization of drought tolerance candidate gene *TaSNAC8‐6A* in wheat.

**Figure S6** Association analysis between InDel‐313 variant‐driven expression of *TaSNAC8‐6A* and the drought tolerant phenotype in wheat.

**Figure S7** Identification and expression analysis of *TaABF* genes in wheat genome.

**Figure S8** Agronomic traits of *Ubi:TaSNAC8‐6A* transgenic wheat plants.

**Figure S9** Transcriptomic analysis of *Ubi:TaSNAC8‐6A* transgenic wheat under well‐watered conditions.

**Table S1** The information of two hundred and sixty NAC super family genes and their corresponding protein sequences in wheat.

**Table S2** Organization of cis‐acting regulatory elements in the promoters of 39 *TaSNAC* genes.

**Table S3** Variations in the *TaSNAC8‐6A* genomic region and their association with wheat drought tolerance.

**Table S4** Significantly up‐ or down‐regulated genes in *Ubi:TaSNAC8‐6A* transgenic wheat grown under well‐watered conditions.

**Table S5** Significantly up‐ or down‐regulated genes in *Ubi:TaSNAC8‐6A* transgenic wheat grown under drought conditions.

**Table S6** NACRS motifs in the promoters of up‐regulated genes involved in ABA‐response, drought stress, and auxin‐response.

**Table S7** Primers used in this research.
