# Supplementary figures and images for: Regulatory changes in TaSNAC8‐6A are associated with drought tolerance in wheat seedlings
Source: Plant Biotechnol J. 2019 Nov 19;18(4):1078–92. doi: 10.1111/pbi.13277 (PMC7061879; doi:10.1111/pbi.13277)

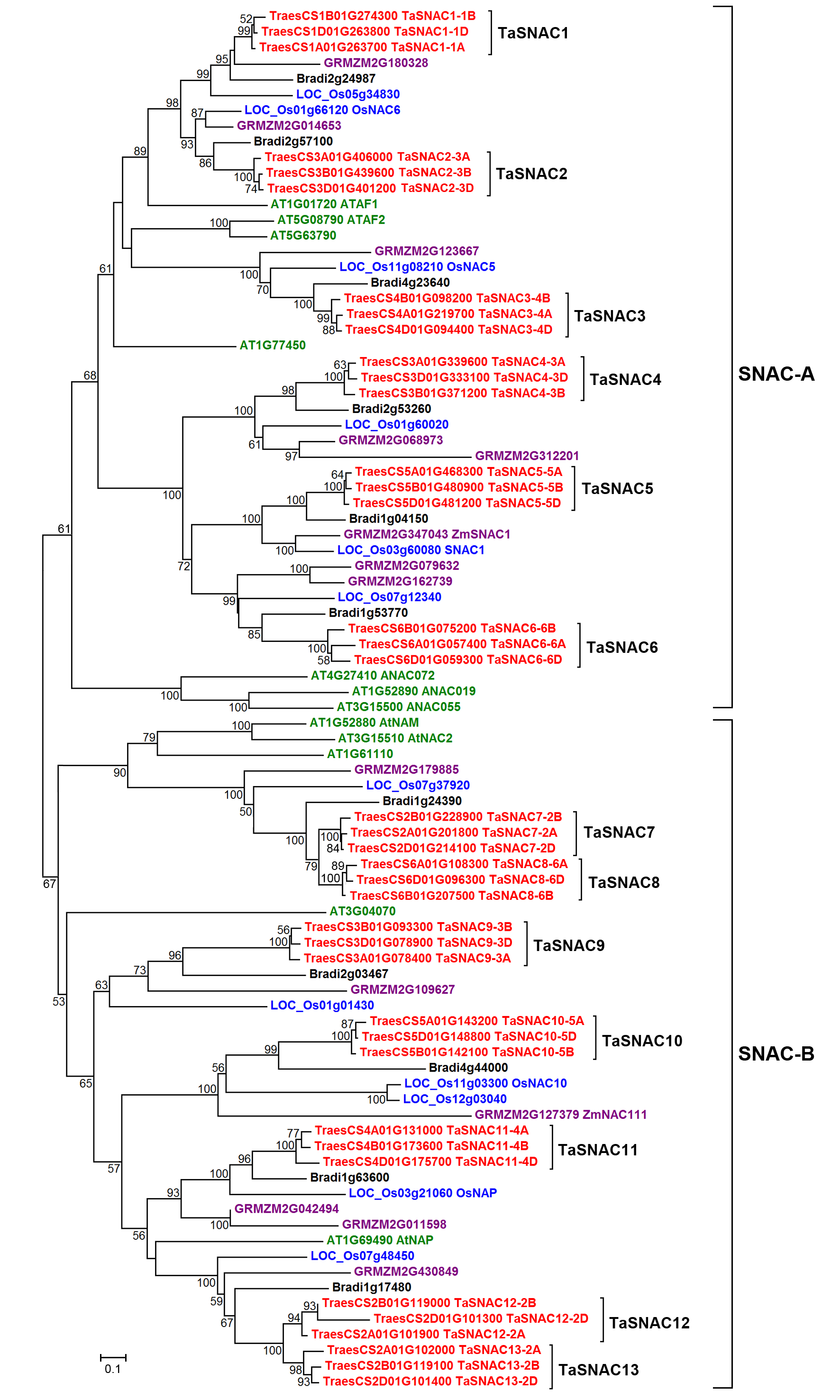

Supplement: Supplementary file 1 — Figure S1 Phylogenetic tree of stress responsive NAC proteins in wheat, rice, B. distachyon, maize, and Arabidopsis. [file PBI-18-1078-s014.tif]

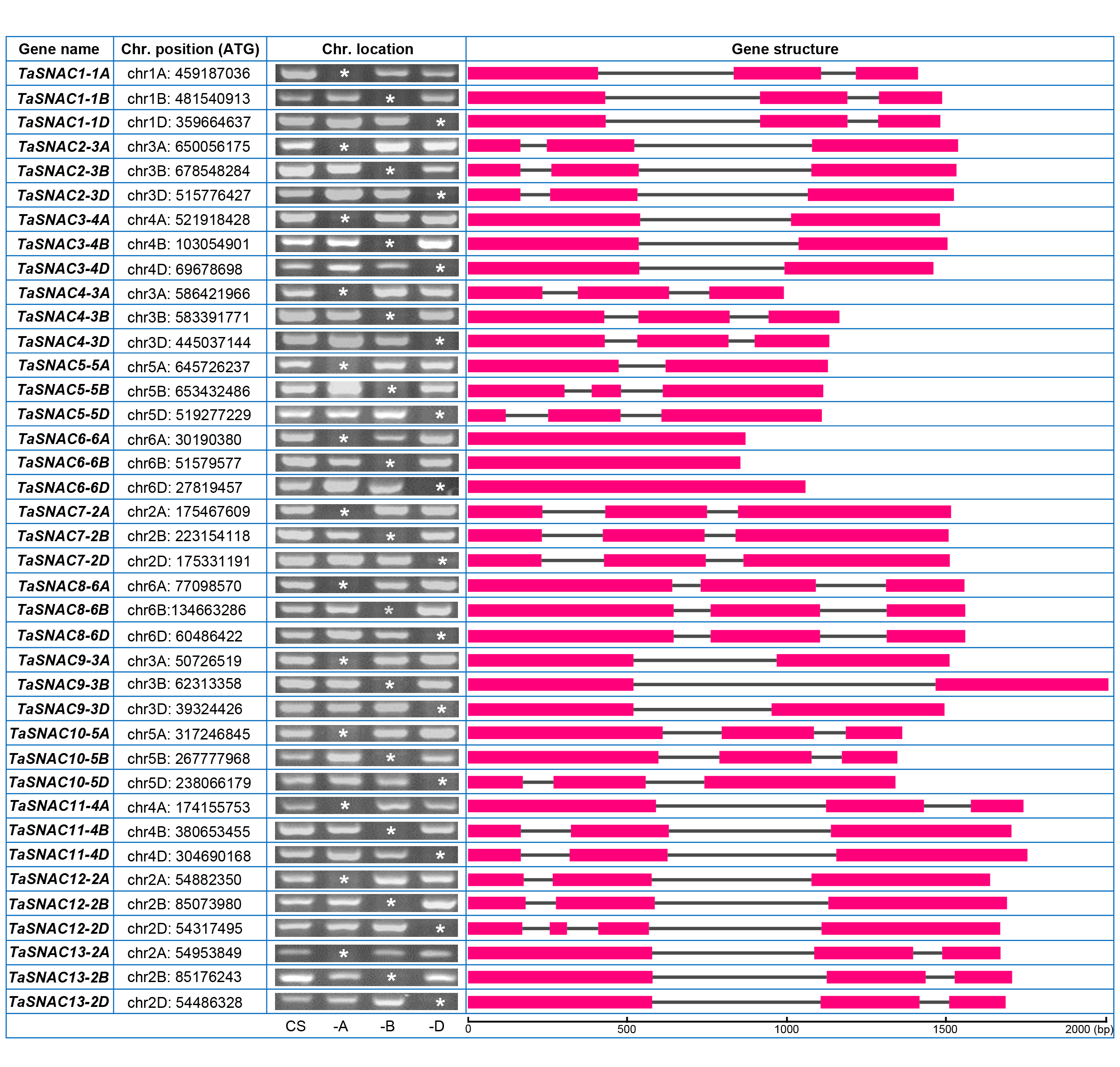

Supplement: Supplementary file 2 — Figure S2 Chromosome location and gene structure analysis of the TaSNAC genes. [file PBI-18-1078-s013.tif]

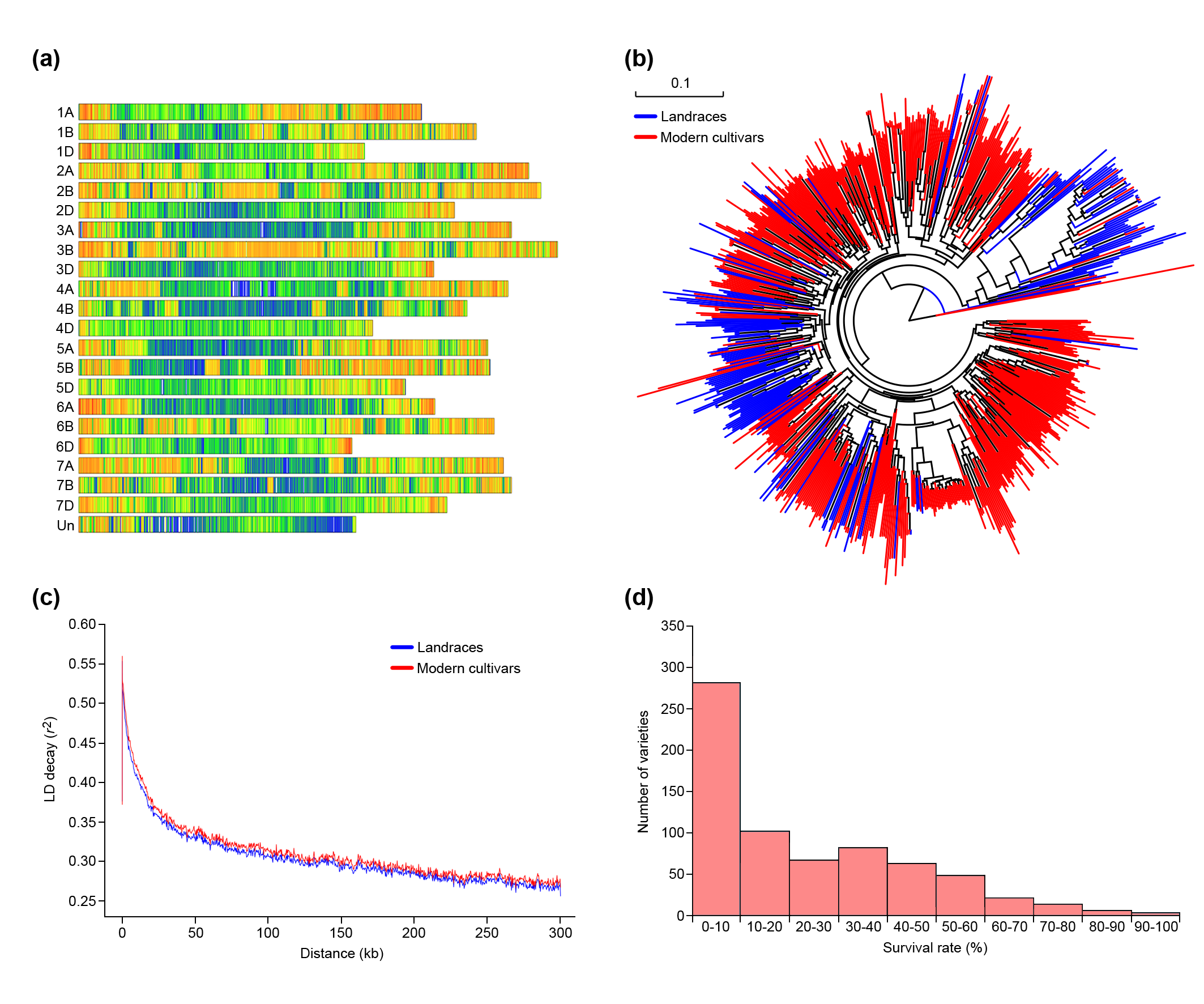

Supplement: Supplementary file 3 — Figure S3 Phylogenetic tree, LD decay, and survival rates of the 700 wheat varieties. [file PBI-18-1078-s015.tif]

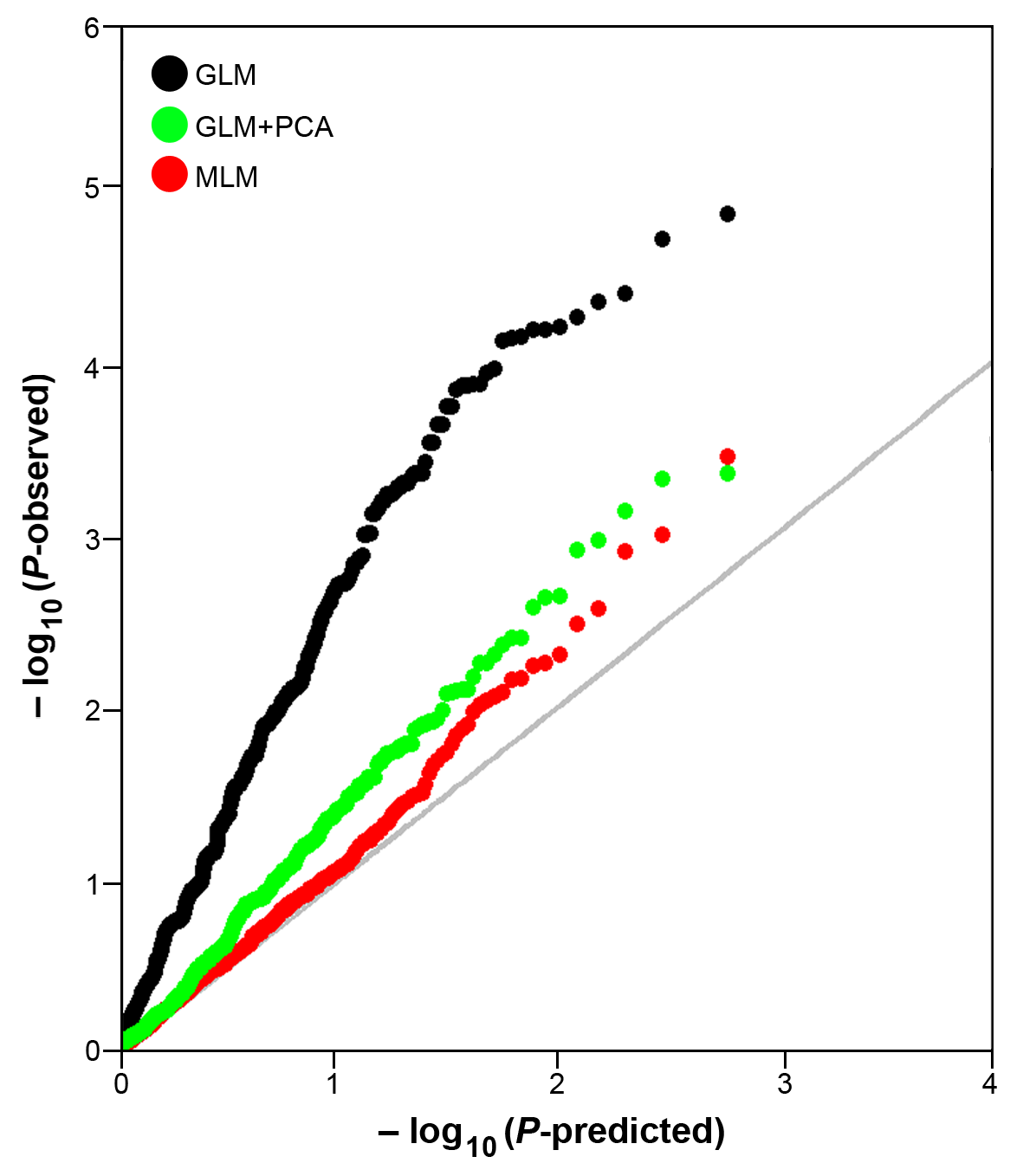

Supplement: Supplementary file 4 — Figure S4 Quantile‐quantile (Q‐Q) plot for association of SNPs and drought tolerance. [file PBI-18-1078-s016.tif]

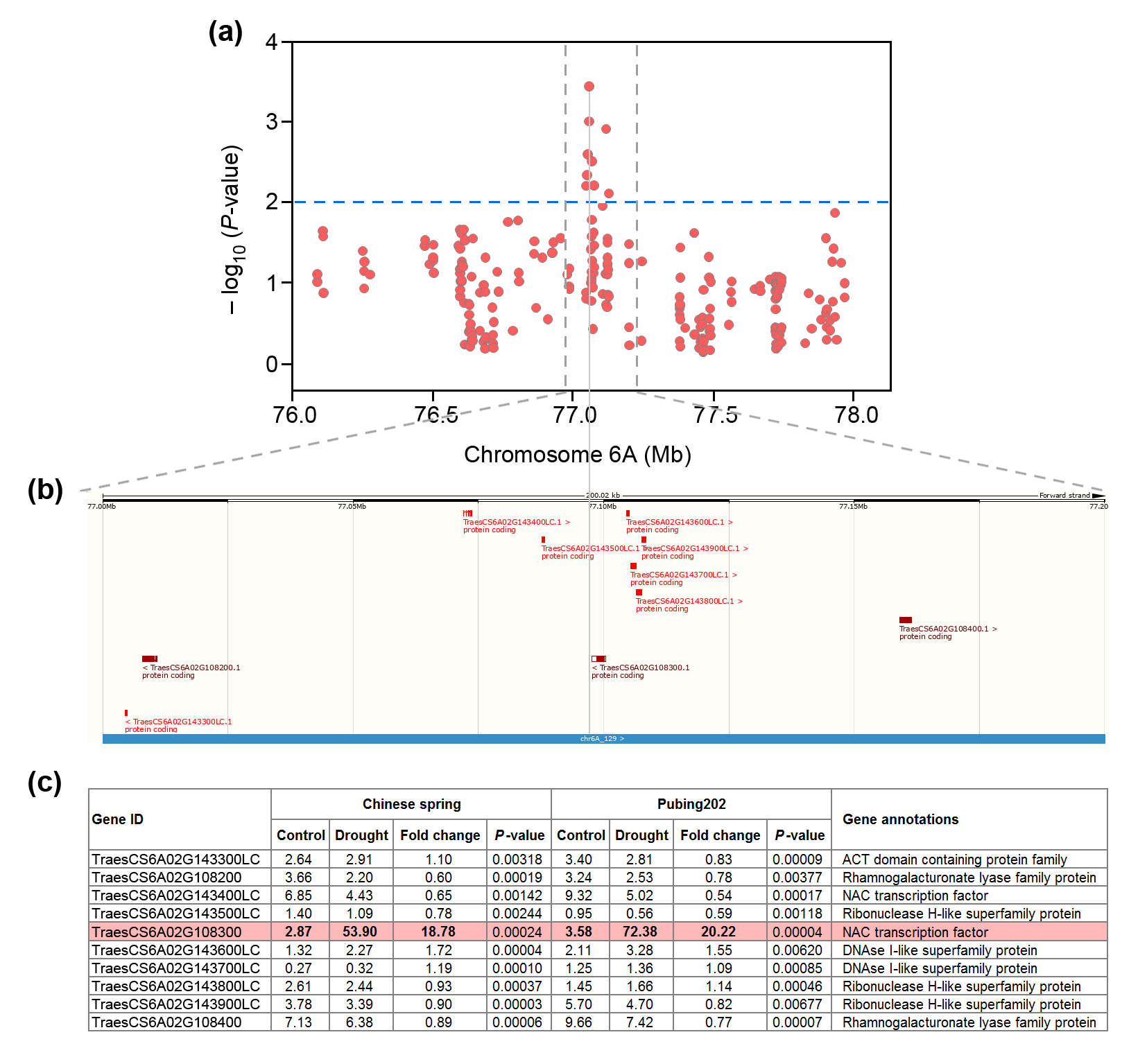

Supplement: Supplementary file 5 — Figure S5 Identification and molecular characterization of drought tolerance candidate gene TaSNAC8‐6A in wheat. [file PBI-18-1078-s002.tif]

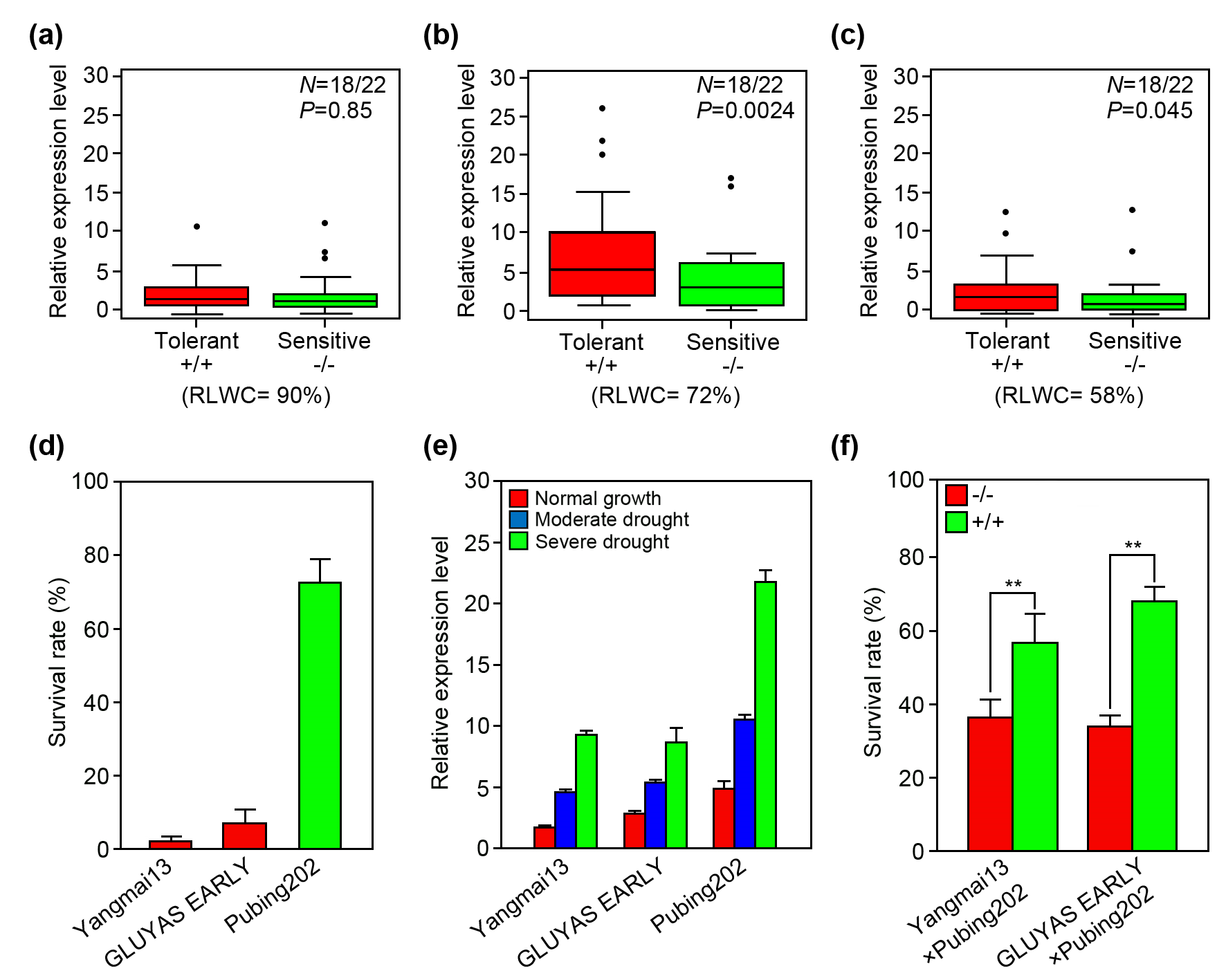

Supplement: Supplementary file 6 — Figure S6 Association analysis between InDel‐313 variant‐driven expression of TaSNAC8‐6A and the drought tolerant phenotype in wheat. [file PBI-18-1078-s001.tif]

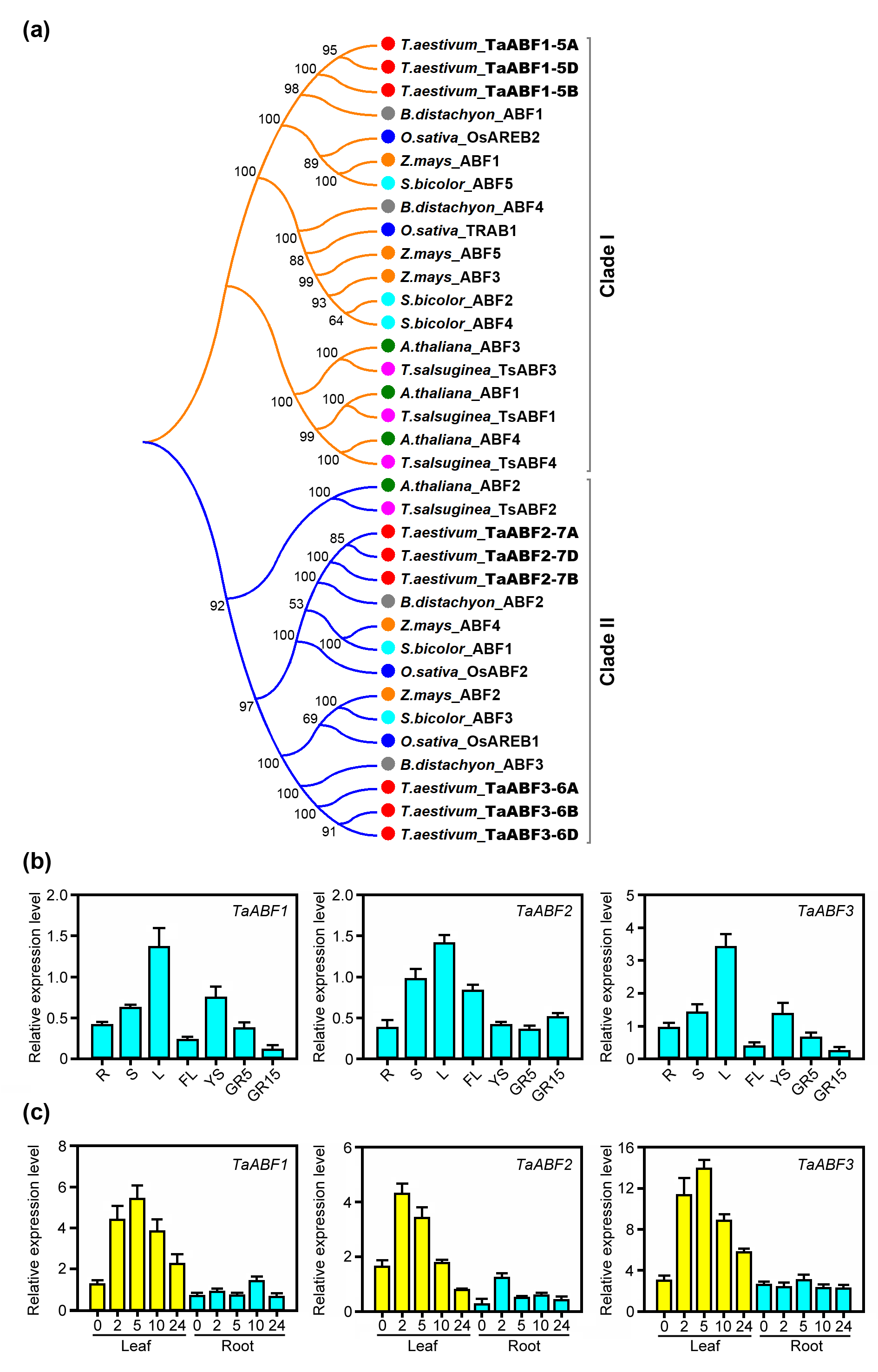

Supplement: Supplementary file 7 — Figure S7 Identification and expression analysis of TaABF genes in wheat genome. [file PBI-18-1078-s003.tif]

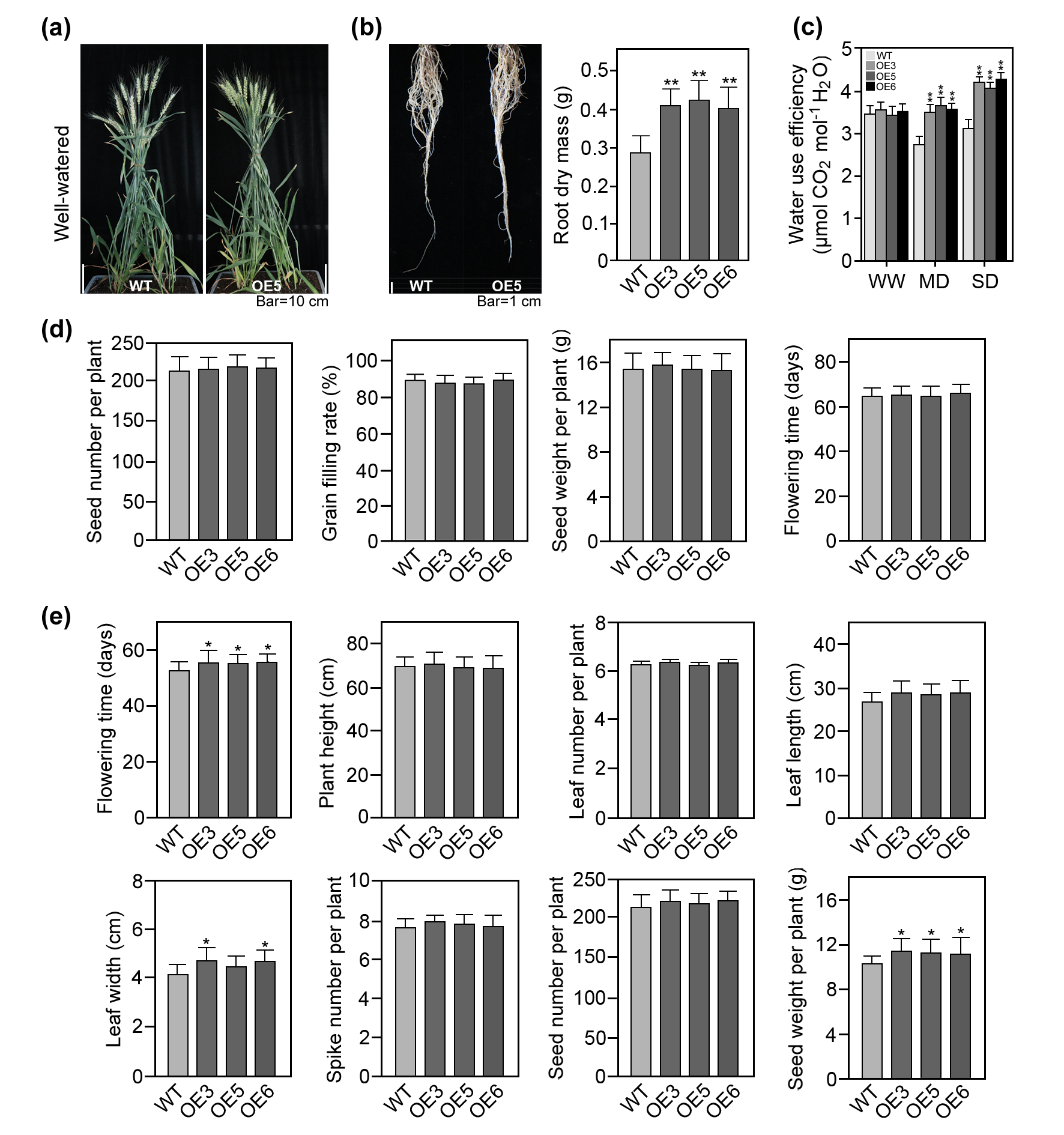

Supplement: Supplementary file 8 — Figure S8 Agronomic traits of Ubi:TaSNAC8‐6A transgenic wheat plants. [file PBI-18-1078-s004.tif]

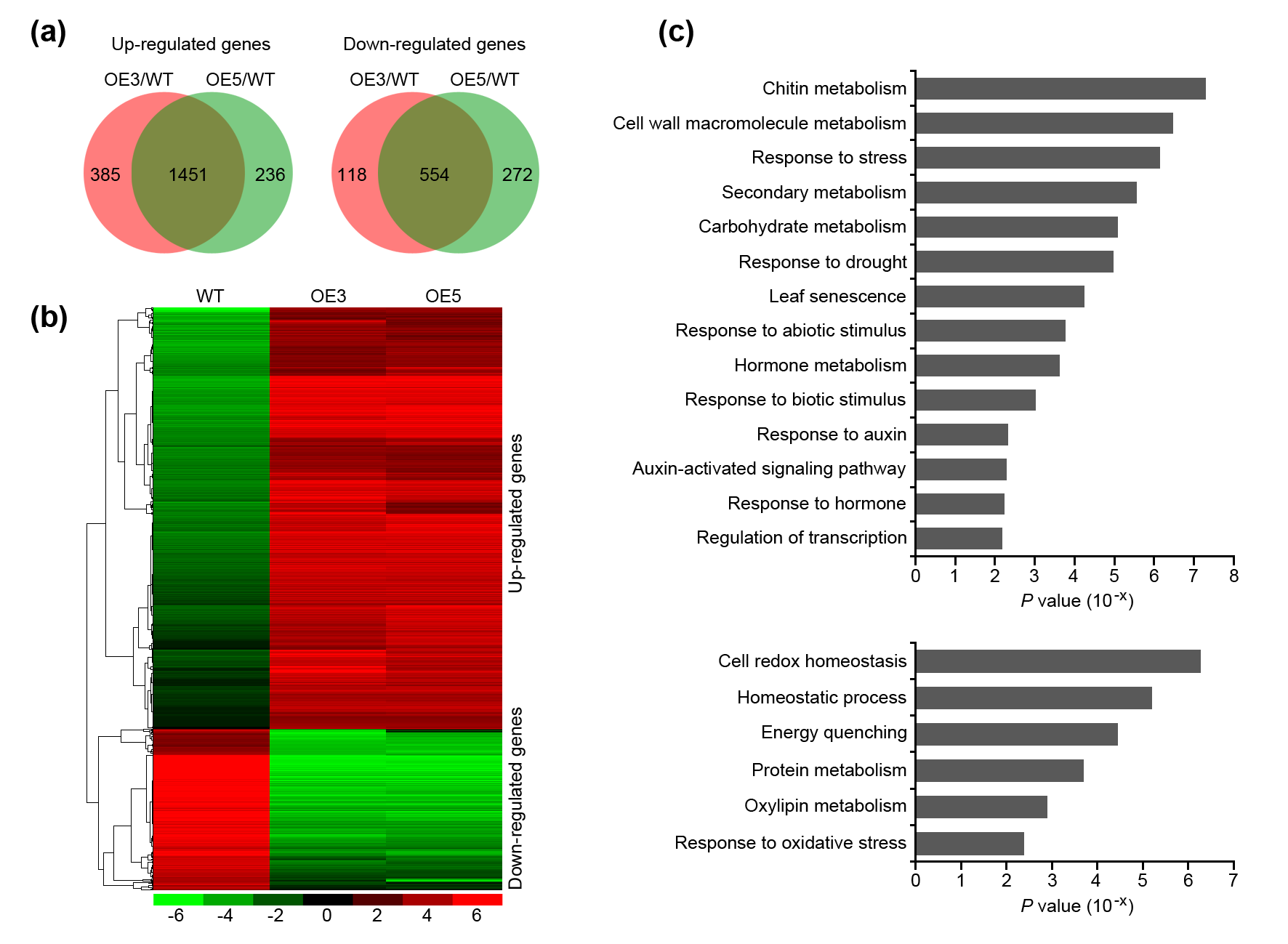

Supplement: Supplementary file 9 — Figure S9 Transcriptomic analysis of Ubi:TaSNAC8‐6A transgenic wheat under well‐watered conditions. [file PBI-18-1078-s005.tif]
